# Stable Zinc Metal Battery Development: Using Fibrous Zirconia for Rapid Surface Conduction of Zinc Ions With Modified Water Solvation Structure

**DOI:** 10.1002/smll.202406481

**Published:** 2024-10-28

**Authors:** Jin Seong Cha, Sanghyeon Park, Yuna Hwang, Eun Jeong Yoon, Donghee Gueon, Jong Min Yuk, Yun‐Chan Kang, Chan‐Woo Lee, Jung Hoon Yang

**Affiliations:** ^1^ Energy Storage Research Department Korea Institute of Energy Research (KIER) Daejeon 34129 Republic of Korea; ^2^ Department of Materials Science and Engineering Korea University Seoul 02841 Republic of Korea; ^3^ Department of Materials Science and Engineering Korea Advanced Institute of Science and Technology Daejeon 34141 Republic of Korea

**Keywords:** dendrite, ion‐transport, solvation structure, surface conduction, zinc‐iodine batteries

## Abstract

The two most critical technical issues in Zn‐based batteries, dendrite formation, and hydrogen evolution reaction, can be simultaneously addressed by introducing negatively charged fibrous ZrO_2_ as a separator. Electron redistribution between ZrO_2_ and Zn^2+^ ions renders the ZrO_2_ surface a preferred adsorption site for Zn^2+^ ions, making surface conduction the primary ion‐transport mode. Surface conduction enables fibrous ZrO_2_ to exhibit a 6.54 times higher single‐Zn‐ion conductivity than that of conventional glass fiber, minimizing the concentration gradient of Zn^2+^ and suppressing dendrite formation. Additionally, strong Zr─O─Zn bonding stabilizes the Zn^2+^ ions with fewer solvated H_2_O molecules (≈2), preventing water molecules from approaching the electrode surface, as evidenced by a 58.8% decrease in the hydrogen evolution rate. Consequently, the cycling stability of a fibrous‐ZrO_2_‐based Zn/Zn symmetric cell (3000 h at 1 mAh cm^−2^ and 5 mA cm^−2^) is approximately ten times greater than that of the conventional variant. Furthermore, a fibrous‐ZrO_2_‐based Zn–I_2_ full cell exhibits a notably high energy density (271.4 Wh kg^−1^) as well as a long lifespan (≈5000 cycles) at an ultrahigh current density (4 A g^−1^).

## Graphical abstract

The fibrous ZrO_2_ continuum is explored as the alternative separator of ZIB. Strong Zn^2+^−ZrO_2_ interaction facilitates surface conduction as the primary mode of ion transport, resulting in a 6.54‐fold increase in single‐Zn‐ion conductivity. The reduced solvation number (≈2) during surface conduction mitigates the HER at the electrode surface, evidenced by a 58.8% reduction in hydrogen generation during cell cycling.

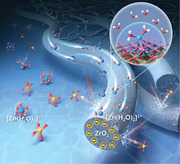

## 1 Introduction

Aqueous Zn‐based batteries, which were pioneered in 1986, are experiencing renewed interest as a cost‐effective, safe alternative to lithium‐ion batteries for realizing next‐generation grid‐scale energy storage systems. Owing to the abundance of Zn in Earth's crust (75 ppm), these batteries have a significantly lower manufacturing cost ($25 per kWh) than that of lithium‐ion batteries ($135 k^−1^Wh).^[^ ^1^ ^]^ Beyond their economic appeal, Zn‐based batteries exhibit noteworthy performance characteristics such as high theoretical capacity (5854 mAh cm^−3^ or 820 mAh g^−1^), low redox potential (−0.76 V versus SHE), and impressive power density owing to their high ionic conductivity (>1 S cm^−1^).^[^ ^2^, ^3^ ^]^ Consequently, aqueous Zn‐based batteries in both flow and static configurations have been realized in combination with various cathodic materials such as MnO_2_, V_2_O_5_, MoO_3_, Prussian blue analogs, Fe, Ce, Cl, Br, and I.^[^ ^4^, ^5^ ^]^

Although Zn‐based batteries show remarkable promise, their development has been consistently hampered by significant challenges. One major issue is the formation of Zn dendrites, which are intricate branching structures primarily caused by inhomogeneities in the electrode surface properties such as the electric field distribution and Zn^2+^‐ion concentration gradient. Uneven electric fields distort the flux direction of Zn^2+^ ions toward protrusions via “tip effects,” promoting localized Zn growth in regions with strong electric fields. Under mass transfer‐limited conditions, where ion transport is slower than electrodeposition, Zn^2+^ ions are rapidly depleted near the electrode surface, creating a significant concentration gradient on the Zn anode.^[^ ^6^, ^7^ ^]^ Upon reaching the electrode surface, Zn^2+^ ions are rapidly electrodeposited, particularly at protrusions, corners, and edges, thereby accelerating the formation of Zn dendrites. These dendrites grow toward the bulk electrolyte and may pierce the separator, causing a short circuit. Another critical challenge is the occurrence of side reactions including the hydrogen evolution reaction (HER) and corrosion. H_2_O molecules in solvated form (that is, [Zn(H_2_O)_6_]^2+^) are transferred from the bulk electrolyte and released on the Zn anode during desolvation, thereby participating in those side reactions.^[^ ^8^ ^]^ Moreover, the HER continuously consumes both Zn metal and water in the electrolyte, lowering the coulombic efficiency (CE) and changing the electrolyte composition. Additionally, the gradual accumulation of hydrogen gas—a side‐reaction byproduct—leads to battery swelling and electrolyte leakage, critically affecting the battery stability. Furthermore, the HER is accompanied by changes in the local pH near the Zn anode, causing the formation of a passivation layer containing zinc hydroxide sulfate (ZHS; Zn_4_SO_4_(OH)_6_•*x*H_2_O),^[^ ^9^, ^10^ ^]^ which increases the surface polarity, further facilitating Zn dendrite growth.

The factors influencing Zn electrodeposition include the electric field, rate of Zn^2+^‐ion transport, and Zn^2+^‐ion solvation structure. To alleviate the inhomogeneity of the electric field on the electrode, electrostatic shielding with co‐cations such as Na^+^, K^+^, Mn^2+^, and Co^2+^ has been adopted as a potent approach.^[^ ^11^, ^12^, ^13^, ^14^ ^]^ Moreover, metal oxides with high dielectric constants (such as ZrO_2_, TiO_2_, SiO_2_, and BaTiO_3_) can interact with the external electric field by polarizing it and generating their own directional electric fields, significantly reducing the inhomogeneity of the electric field on Zn anode.^[^ ^15^, ^16^, ^17^, ^18^, ^19^ ^]^ Additionally, electrode modification with highly conductive materials (such as graphite oxide (GO) and MXenes) can achieve a uniform charge distribution across the surface of the Zn anode.^[^ ^20^, ^21^ ^]^ Substantial efforts have also been devoted to enhancing the rate of Zn^2+^‐ion transport. For instance, 3D anode hosts with high specific areas alleviated the Zn^2+^ concentration polarization by exhibiting a low areal current density.^[^ ^22^, ^23^ ^]^ Moreover, electrolyte stirring has helped accelerate Zn^2+^‐ion transport.^[^ ^24^ ^]^ The stirring‐induced forced convection of the electrolyte augments the diffusion and migration of Zn^2+^ ions, thereby ameliorating concentration polarization. Additionally, anode surface modification has recently been found to promote Zn^2+^ migration through the coating layer with zincophilic functional groups.^[^ ^25^, ^26^ ^]^ In terms of attempts made to regulate the [Zn(H_2_O)_6_]^2+^ solvation structure, electrolyte engineering with highly concentrated electrolytes,^[^ ^27^ ^]^ ionic liquids,^[^ ^28^ ^]^ deep eutectic electrolytes,^[^ ^29^ ^]^ and weakly solvating electrolytes^[^ ^30^ ^]^ has shown potential for weakening the Zn^2+^ ion–H_2_O bond strength. Additionally, the introduction of certain functional additives with a high affinity toward Zn species has helped adjust the [Zn(H_2_O)_6_]^2+^ solvation structure.^[^ ^31^, ^32^ ^]^ However, because these strategies focus on individual performance characteristics, they cannot fully resolve the issues related to all three influencing factors. For example, while concentrated electrolytes are highly effective in altering the solvation structure and suppressing the HER, they deteriorate the Zn^2+^‐ion mass transfer characteristics by increasing viscosity and reducing bulk conductivity.

**Figure 1:**
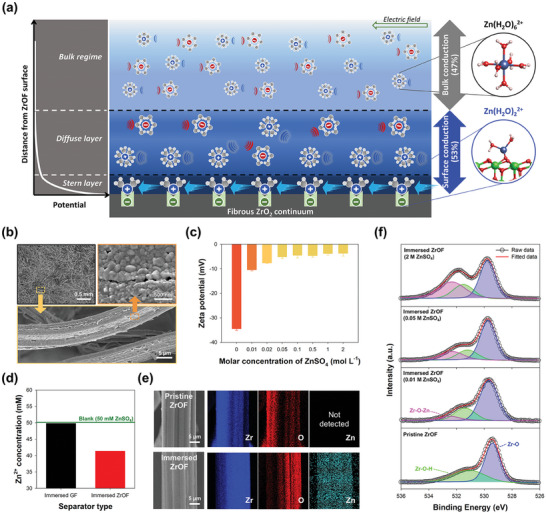
Zn^2+^‐interacting ability and physicochemical properties of ZrO_2_ felt (ZrOF) surface. a) Schematic illustrating diffusion, migration, and surface conduction of Zn^2+^ ions on ZrOF surface. b) SEM images of ZrOF surface. c) Variation in zeta potential of ZrOF powder dispersed in ZnSO_4_ solutions with different molar concentrations. d) Change in concentration of Zn^2+^ ions in 50 mm ZnSO_4_ solution after immersion of glass fiber (GF) or ZrOF, as determined by ICP‐OES analysis. e) High‐magnification SEM images and corresponding EDX‐spectroscopy‐derived elemental maps. f) High‐resolution O 1*s* spectra deconvoluted from the XPS survey spectrum.

Surface conduction is an electrokinetic phenomenon in which ions move along a charged surface in the direction of an electric field.^[^ ^33^, ^34^ ^]^ For example, cations preferentially adsorb and distribute on a negatively charged surface, and ion migration begins when the electric field exceeds the adsorption strength of these cations on the surface. As indicated by Lyklema, surface conduction in the stern layer notably enhances the rate of cation transport on a negatively charged surface.^[^ ^35^ ^]^ Therefore, the present study was aimed at investigating the surface conduction of Zn^2+^ ions on a fibrous ZrO_2_ continuum to simultaneously address the aforementioned critical hindrances. A ZrO_2_ felt (ZrOF) scaffold, which comprised interconnected ZrO_2_ fibers with a negatively charged surface,^[^ ^36^, ^37^, ^38^ ^]^ served as a potent alternative to the conventional glass fiber (GF) separator used in Zn‐based battery systems. Notably, its negatively charged surface functioned as a rapid Zn^2+^‐ion transport pathway through electrostatic adsorption. The Zn^2+^‐ion transport through the ZrOF occurred in three modes: diffusion, migration, and surface conduction (**Figure** **1**a). While diffusion and migration occurred in the bulk electrolyte surrounding the ZrOF, surface conduction transpired within the interfacial double layer between the ZrOF and electrolyte, where Zn^2+^ ions were more densely distributed than in the bulk owing to electrostatic attraction. Specifically, investigations were conducted to determine the extent to which the surface conduction on the fibrous ZrO_2_ continuum helped boost Zn^2+^‐ion transport and thereby inhibit Zn dendrite formation. The surface conduction altered the Zn^2+^‐ion solvation structure through a reduction in the H_2_O coordination number. This minimized the water molecule access to the electrode surface, preventing the HER and corrosion. Computational analysis corroborated the decrease in the H_2_O coordination number by demonstrating the stabilization of Zn^2+^ ions on the ZrO_2_ surface, which resulted in a modified [Zn(H_2_O)_2_]^2+^ solvation structure. Consequently, a Zn–I_2_ battery with the ZrOF separator exhibited an impressively long lifespan of ≈5000 cycles, even at an ultrahigh current density of 4 A g^−1^ (equivalent to an areal current density of 51.5 mA cm^−2^) and a high capacity of 181.6 mAh g^−1^.

## 2 Results and Discussion

### 2.1 Interactions between Zn^2+^ Ions and Negatively Charged ZrOF Surface

For effective surface conduction, the electric field lines running from one adsorption site to the next should be in conductive contact, for example, in the form of the fibrous continuum exhibited by the ZrOF. To validate these aspects, the surface and cross‐sectional morphologies of the ZrOF were examined by field‐emission scanning electron microscopy (FE‐SEM) (Figure 1b; Figure S1, Supporting Information). The results revealed a free‐standing porous structure with interconnected pore channels featuring an ion pathway with bulk conductivity *σ* ^b^ through the filling electrolyte. The individual ZrO_2_ fibers can facilitate ion conduction with surface conductivity *σ* ^s^. The X‐ray diffractometry (XRD) pattern of the ZrOF was indexed to the cubic crystal structure of ZrO_2_ (JCPDS # 49–1642) as the prevailing phase (Figure S2, Supporting Information).

To quantitatively analyze the surface charge of the ZrOF, the zeta potential of the ZrOF was measured after dispersing it in solutions with different molar concentrations of ZnSO_4_ (Figure 1c; Figure S3, Supporting Information). While the zeta potential was highly negative in pure water (−34.5 mV), it sharply increased to −5.2 mV as the molar concentration of ZnSO_4_ increased to 50 mm. This positive shift in zeta potential was associated with the effective adsorption of Zn^2+^ ions on the ZrOF surface via electrostatic attractive interactions.^[^ ^39^, ^40^, ^41^ ^]^ Therefore, even at small amounts, the Zn^2+^ ions preferentially adsorbed on the ZrOF surface rather than being present in the bulk solution. To estimate the amount of adsorbed Zn^2+^ ions after immersing a ZrOF fragment (2.5 g) in a 50 mm ZnSO_4_ solution, the change in concentration of Zn^2+^ ions in the solution was measured by inductively coupled plasma‐optical emission spectrometry (ICP‐OES) and compared with that of a GF fragment (Figure 1d). The results suggested that the Zn^2+^ ions hardly adsorbed on the GF specimen and remained in the bulk solution. In contrast, the Zn^2+^ concentration of the ZrOF‐containing solution decreased from 50 to 41 mm, indicating that the negatively charged ZrOF surface provided favorable sites exhibiting strong electrostatic attraction to Zn^2+^ ions. Additionally, the Zn^2+^ ions adsorbed on the ZrOF surface were visualized by elemental mapping conducted through energy‐dispersive X‐ray (EDX) spectroscopy (Figure 1e), which revealed the uniform distribution of Zn on the ZrOF surface.

X‐ray photoelectron spectroscopy (XPS) was performed to identify changes in the surface composition and binding structure of the ZrOF upon immersion in a ZnSO_4_ solution whose concentration was varied. The overall XPS survey spectrum (Figure S4, Supporting Information) certified the presence of Zr, O, and Zn on the ZrOF surface. The O 1*s* spectra showed an asymmetric peak that could be divided into two parts corresponding to lattice oxygen and surface oxygen at low and high energies, respectively (Figure 1f).^[^ ^42^, ^43^, ^44^ ^]^ The lattice oxygen peak centered at 529.6 eV was related to O^2−^ ions in the cubic ZrO_2_ structure (that is, the Zr─O peak), whereas the surface oxygen peaks at 531.3 and 532.4 eV corresponded to Zr─O─H and Zr─O─Zn, respectively. As the molar concentration of ZnSO_4_ increased, the Zr─O─Zn functional group became increasingly more abundant, implying a strong affinity between Zn^2+^ ions and the surface oxygen of cubic ZrO_2_, thereby promoting surface conduction. The Zr─O─Zn interactions also caused a change in the oxidation state of Zr, increasing the portion of Zr^(4−δ)+^ (δ > 0), as revealed in the high‐resolution Zr 3*d* spectra (Figure S5, Supporting Information).^[^ ^45^, ^46^ ^]^ Specifically, the Zr─O peak was deconvoluted into two regions: the typical Zr^4+^ oxidation state and a partially reduced Zr^(4−δ)+^ (δ > 0) variant.^[^ ^45^, ^46^ ^]^ The electrostatic interactions between Zn^2+^ ions and the ZrOF were corroborated by high‐resolution Zn *2p* spectra (Figure S6, Supporting Information). When the ZrOF was immersed in the ZnSO_4_ solution, a positive shift in the Zn^2+^ 2*p* peaks was observed, presumably owing to electron exchange between Zn^2+^ and electron‐rich Zr─O groups.^[^ ^45^, ^47^ ^]^ The dominant peak shift occurred at Zn^2+^ concentrations below 0.05 m, aligning with the zeta potential findings. These results conclusively affirmed the interactions between Zn^2+^ ions and the negatively charged ZrOF surface, indicating efficient binding for surface conduction.

### 2.2 Surface‐Conduction‐Driven Transport of Zn^2+^ Ions on ZrOF Separator

**Figure 2:**
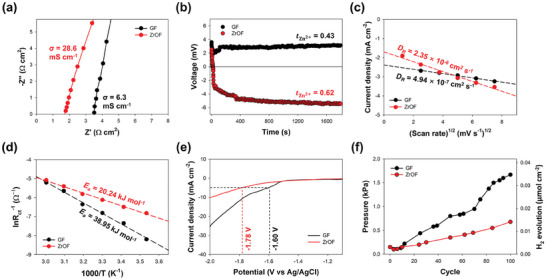
Electrochemical characterization of GF and ZrOF separators (black and red datapoints, respectively). a) Ionic conductivities calculated by EIS analysis performed using a symmetric stainless steel cell. b) Zn^2+^‐ion transference numbers are determined by measuring the liquid junction potential. c) Diffusion coefficients obtained from CV curves acquired at different scan rates. d) Arrhenius profiles obtained from Nyquist plots at different temperatures and corresponding activation energies. e) LSV curves for the hydrogen evolution reaction (HER) at a scan rate of 10 mV s^−1^. f) Quantitative measurement of pressure and corresponding hydrogen evolution as a function of cumulative cycles for Zn symmetric cell at 1 mAh cm^−2^ and 1 mA cm^−2^.

To explore the electrochemical behavior of Zn^2+^ ions on the interface between the negatively charged separator and the electrolyte (that is, the 2 m ZnSO_4_ solution commonly used in Zn‐based batteries), the ionic conductivity, transference number, and activation energy for Zn^2+^‐ion desolvation were investigated for the GF and ZrOF separators. Ionic conductivity was determined by electrochemical impedance spectroscopy (EIS) using a symmetric cell with a stainless steel current collector (**Figure** **2**a). The ionic conductivity for the ZrOF separator (28.6 mS cm^−1^) was considerably higher than that for the GF separator (6.3 mS cm^−1^) presumably owing to the rapid surface‐conduction‐guided Zn^2+^‐ion transport on the ZrOF separator. To determine the contribution of a single Zn^2+^ ion on the total ionic conductivity or calculate the Zn^2+^‐ion transference number ($t_{Zn^{2+}}$), the liquid junction potential was monitored as a function of time (Figure 2b; Section S2, Supporting Information). The $t_{Zn^{2+}}$ value for the ZrOF separator (0.62) was notably higher than that for the GF equivalent (0.43). The transference number for aqueous Zn sulfate solutions has been reported to be ≈0.40;^[^ ^48^, ^49^, ^50^ ^]^ this value is similar to that for the GF separator in the present study, which indicates that most Zn^2+^ ions were transported through the GF separator in the same way as in the bulk solution—that is, by diffusion and migration. In contrast, the improved $t_{Zn^{2+}}$ value of the ZrOF separator implies that the ion transport differed from that in the conventional bulk solution, evidently owing to the surface conduction of Zn^2+^ ions through the electrolyte–ZrO_2_ fiber interface. The negatively charged ZrOF surface selectively transported only Zn^2+^ cations rather than SO_4_^2−^ anions, increasing $t_{Zn^{2+}}$. Consequently, the single‐Zn^2+^‐ion conductivity was calculated by multiplying the total ionic conductivity and Zn^2+^‐ion transference number. The single‐Zn^2+^‐ion conductivity for the ZrOF separator (17.7 mS cm^−1^) was ≈6.54 times higher than that for the GF variant (2.7 mS cm^−1^). Additionally, for the ZrOF separator, the contribution of surface conduction to the overall single‐Zn^2+^‐ion conductivity was 53.2% (Section S3, Supporting Information), underscoring the potency of the negatively charged surface in facilitating Zn^2+^‐ion conduction across the ZrOF separator.

The enhancement in the mass transfer rate of Zn^2+^ ions by the negatively charged separator was confirmed by comparing diffusion coefficients. Cyclic voltammetry (CV) analysis was performed using a 20 mm ZnSO_4_ solution on a carbon electrode at various scan rates. Based on the Randles–Ševčík relationship, the ZrOF separator achieved a significantly higher diffusion coefficient (2.4 × 10^−6^ cm^2^ s^−1^) than that of the GF separator (4.9 × 10^−7^ cm^2^ s^−1^) (Figure 2c; Figures S7, S8, Supporting Information). Considering the acceleration of dendrite formation by the rapid depletion of Zn^2+^ ions near the electrode surface,^[^ ^6^, ^7^ ^]^ the enhanced mass transfer characteristics through the ZrOF helped achieve dendrite‐free electrodeposition. Moreover, chronoamperometry (CA) tests were performed at a constant overpotential of 400 mV because the change in current could sensitively reflect the 2D‐diffusion‐induced variation in the electrode surface morphology (Figure S9, Supporting Information). Notably, the high mass transfer rate of Zn^2+^ ions through the ZrOF separator prevented the adsorbed Zn^2+^ ions from experiencing 2D diffusion and developing into dendritic growth.

The charge transfer resistance was measured by EIS analysis at different temperatures from 10 to 60 °C, and the activation energy *E*_a_ for the charge‐transfer reaction was calculated using the Arrhenius equation (Figure 2d; Figure S10, Supporting Information).^[^ ^51^, ^52^ ^]^ The ZrOF separator achieved a significantly lower activation energy (20.2 kJ mol^−1^) than that of the GF separator (38.9 kJ mol^−1^). Considering the anode consistency of zinc foil, the difference in activation energy likely originated from changes in the solvation structure of Zn^2^⁺ ions after they reached the electrode surface. As discussed below, the strong interactions between the negatively charged ZrOF and Zn^2+^ ions altered the Zn^2+^‐ion solvation structure from [Zn(H₂O)_6_]^2+^ to [Zn(H₂O)_2_]^2+^. The lowered desolvation energy led to homogeneous Zn electrodeposition with a reduced nucleation overpotential (NOP). CV profiles for Zn deposition/stripping were acquired at a scan rate of 20 mV s^−1^ (Figure S11, Supporting Information). In this analysis, the deposition potential was considered the point at which Zn^2+^ ions began to be reduced on the substrate during the forward potential scan (A), and the crossover potential was considered the intersection point at which the current switched from the cathodic to the anodic value during the reverse potential scan (B). The difference between these two potentials (|A − B|) indicated the extent of polarization from Zn nucleation, or NOP.^[^ ^53^, ^54^ ^]^ The NOP for Zn^2+^ ions transported through the ZrOF separator (51.9 mV) was approximately half that for the GF separator (95.1 mV), suggesting that the use of the ZrOF separator promoted the reversibility of the Zn^0^/Zn^2+^ redox reaction. Furthermore, the cathodic peak current density for the ZrOF (8.7 mA cm^−2^) was higher than that for the GF variant (4.2 mA cm^−2^) owing to the enhanced mass transfer of Zn^2+^ ions through the ZrOF separator and the lowered desolvation energy.

The Zn^2+^‐ion solvation structure is closely related to the HER because coordinated water molecules can approach the electrode surface and be reduced to hydrogen gas.^[^ ^55^, ^56^ ^]^ To qualitatively evaluate the effects of the ZrOF separator on the HER kinetics, linear sweep voltammetry (LSV) was performed using a Zn^2+^‐ion‐free 1 m Na_2_SO_4_ electrolyte (Figure 2e). The cathodic current for the HER with the GF separator exceeded −5 mA cm^−2^ at −1.60 V, whereas that with the ZrOF separator exhibited a comparable value at a potential below −1.78 V. Additionally, the slope of the HER‐related Tafel polarization curve for the ZrOF separator (548.1 mV dec^−1^) surpassed that for the GF separator (406.5 mV dec^−1^) (Figure S12, Supporting Information), indicating that the HER was suppressed in the case of the ZrOF separator. Importantly, the hydrogen evolution was quantitatively measured by monitoring the internal pressure of the Zn symmetric cell as a function of cycle number (Figure 2f) and setting the cutoff capacity and current density to 1 mAh cm^−2^ and 1 mA cm^−2^, respectively. Overall, the ZrOF separator exhibited significantly less hydrogen evolution than that of the GF separator. After 100 cycles, the total amount of hydrogen generated by the ZrOF separator (0.014 µmol cm^−2^) was 58.8% less than that of the GF separator (0.034 µmol cm^−2^). Therefore, the HER suppression by the ZrOF separator prevented local pH changes in the electrolyte, further inhibiting the formation of the passivation layer. To determine the extent of corrosion, Tafel polarization curves were obtained using Zn^2+^‐ion‐containing 2 m ZnSO_4_ as the electrolyte (Figure S13, Supporting Information). The ZrOF separator exhibited a higher corrosion potential and lower corrosion current density than those of the GF separator, indicating suppression of corrosion.

Overall, these findings demonstrate that the ZrOF separator offers advantages such as high single‐Zn^2+^‐ion conductivity, enhanced Zn^2+^‐ion mass transfer, low desolvation energy, and reduced hydrogen generation in Zn‐based batteries.

### 2.3 Energetics of Hydrated Zn^2+^ Ions on ZrOF Surface

To further investigate the surface conduction of Zn^2+^ ions on the ZrOF, the solvated structures of Zn^2+^ ions on the ZrO_2_ (111) surface and in the bulk solution were simulated using the density functional theory (DFT) and ab initio molecular dynamics (AIMD) (see Computational Details in Section S1, Supporting Information).

**Figure 3:**
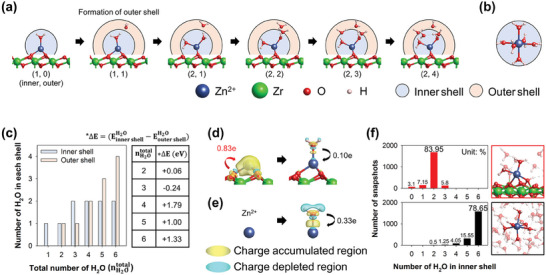
DFT‐ and ab initio molecular dynamics (AIMD)‐derived model of Zn^2+^ ion solvation on ZrO_2_ surface and in bulk solution. a) Thermodynamically stable Zn^2+^ ion solvation structures on ZrO_2_ (111) with sequential adsorption of H_2_O. b) Zn^2+^‐ion solvation structure in bulk solution. c) Distribution of H_2_O molecules in inner and outer shells with respect to the total number of H_2_O molecules (left), and energy differences at the positions of the newly adsorbed H_2_O molecules (right). Charge density difference maps of initial stabilization of Zn^2+^ ion on d) ZrO_2_ and e) in bulk solution; the yellow and cyan regions indicate charge accumulated and charge depleted zones, respectively (isosurface level: 0.01 e Å^−3^). f) Distribution of AIMD snapshots against a number of H_2_O molecules in the inner shell for ZrO_2_ and bulk solution.

Sequential hydration of a Zn^2+^ ion on the ZrO_2_ surface with up to six H_2_O molecules was studied by comparing the energetics and geometry for identical modes of coordination with H_2_O in two solvation configurations: inner and outer shells (**Figure** **3**a; Figure S14, Supporting Information). H_2_O molecules in the inner and outer shells were considered to form direct and indirect bonds, respectively, with the Zn^2+^ ion. The two shells, inner and outer, are distinguished by the radius of the Zn‐water complex (2.8 Å), where Zn^2+^ ion is coordinated with six H_2_O molecules in the bulk solution (Figure 3b).

In the scenario with the ZrO_2_ surface, the maximum coordination number (CN) of Zn^2+^ ions with H_2_O in the inner shell was lower than that of Zn^2+^ ions in the bulk solution (two and six, respectively). When the number of H_2_O molecules was increased to two (Figure 3c), the newly adsorbed H_2_O (total number of H_2_O units, $n_{H2O}^{\mathrm{total}}=2)$ remained stable in the outer shell, despite the miniscule formation energy difference between the two shells $\left(\Delta E=EE_{\mathrm{inner}\;\mathrm{shell}}^{H_{2}O}-E_{\mathrm{outer}\;\mathrm{shell}}^{H_{2}O}=0.06\;eV\right)$. After the third H_2_O molecule $\left(n_{H2O}^{\mathrm{total}}=3\right)$ occupied the inner shell, thereby achieving CN = 2, all subsequent H_2_O molecules $\left(n_{H2O}^{\mathrm{total}}\;\geq \;4\right)$ inhabited the outer shell with high stability. The lower maximum CN of the Zn^2+^ ion with H_2_O on the ZrOF than that in the bulk solution was due to the significant stabilization of Zn^2+^ ions on the ZrO_2_ surface, induced by substantial electron redistribution upon Zn^2+^‐ion adsorption on the ZrO_2_ surface (adsorption energy = −23.44 eV).

The electron redistribution was visualized by mapping the charge density difference before and after the Zn^2+^–H_2_O interactions on the ZrO_2_ surface and in the bulk solution (Figure 3d,e, respectively). The adsorbed Zn^2+^ ion was stabilized by significant electron transfer from the ZrO_2_ surface to the Zn^2+^ ion (0.83e). Consequently, the stabilization by H_2_O binding (0.10e) was less significant than that in the bulk solution (0.33e). This implies that the adsorbed Zn^2+^ ion was already substantially stabilized by its interaction with the ZrO_2_ surface, rendering additional stabilization by H_2_O molecules unnecessary, as evidenced by the reduced maximum CN of the Zn^2+^ ion.

The distribution of 2000 snapshot structures obtained from AIMD simulations of Zn^2+^‐ion solvation in an ambient H_2_O environment was evaluated for the ZrO_2_ surface and in the bulk solution (Figure 3f). For each snapshot structure, H_2_O molecules in the inner shell were counted when the bond length between Zn^2+^ ions and the oxygen of H_2_O was less than 2.8 Å, similar to the DFT calculations, with the number of H_2_O molecules in the inner shell representing the CN of Zn^2+^ ions with H_2_O. On the ZrO_2_ surface, structures with two H_2_O molecules in the inner shell were dominant (83.95%), whereas in the bulk solution, structures with six H_2_O molecules in the inner shell were most common (78.65%), with some structures having five H_2_O molecules also observed (15.55%).

To obtain more direct evidence of the solvation structure around Zn^2+^ ions, we operated a Zn//Zn symmetric flow cell (Figure S15, Supporting Information). In this cell, a cation exchange membrane (Nafion NR‐211) was used as a separator to restrict the movement of free water between the anode and cathode while allowing selective permeation of Zn^2+^. The transport of Zn^2+^ ions, accompanied by solvated H₂O molecules, cause changes in the electrolyte volume at both the anode and cathode. Specifically, as the solvation number increases, the number of water molecules migrating through the membrane also increases proportionally, resulting in a more significant change in electrolyte volume. For the GF separator, a volume of 10.8 mL was increased in the anolyte and decreased in the catholyte, indicating that this volume of electrolyte was transported during the passage of 65.3 × 10^−3^ moles of Zn^2+^ ions through the membrane (Figure S15c, Supporting Information). Considering the electrolyte density, concentration, and volume of the Zn deposition layer, this volume change corresponds to 8.96 water molecules per Zn^2+^ ion. Typically, Zn^2+^ ions are well known to be solvated by six water molecules in the bulk electrolyte. Given that GF exhibits similar transport characteristics to the bulk electrolyte, the excess of 2.96 water molecules (i.e., beyond the 6 initially associated with the Zn^2+^ ions) can be attributed to the transport of free water molecules through the NR‐211. The ZrOF membrane exhibited a significantly smaller volume change of 7.2 mL compared to the GF membrane, which corresponds to 4.98 water molecules per Zn^2+^ ion (Figure S15d, Supporting Information). The smaller electrolyte volume change with the ZrOF separator suggests that the strong interaction between Zn^2+^ ions and the negatively charged ZrOF surface affects the solvation behavior of Zn^2+^ ions, reducing the H_2_O coordination number. Assuming a similar level of transport contribution by free water molecule as observed with GF, the solvation number of Zn^2+^ ions for the ZrOF separator can be calculated as 2.02. This result aligns consistently with the solvation number obtained from DFT and AIMD calculations and serves as key evidence of ZrOF‐induced changes in the solvation structure. Furthermore, the shift in the *v*(SO_4_^2−^) band in the Raman spectra indicates that ZrOF affects the solvation structure by reducing the number of H_2_O molecules coordinated around Zn^2+^ ions (Figure S16, Supporting Information).

### 2.4 Galvanostatic Deposition/Stripping Behavior of Zn with ZrOF Separator

**Figure 4:**
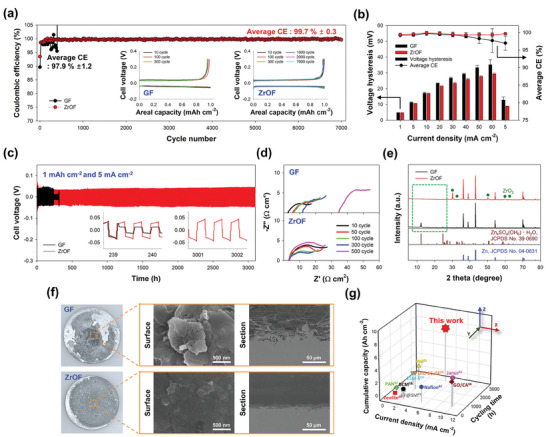
Reversibility and cycling stability of galvanostatic Zn deposition/stripping with ZrOF separator. a) Coulombic efficiency (CE) of carbon/Zn asymmetric cells at an areal capacity of 1 mAh cm^−2^ and current density of 5 mA cm^−2^. b) Variations in voltage hysteresis and average CE at different current densities (1–60 mA cm^−2^). c) Voltage–time profiles for Zn symmetric cells at an areal capacity of 1 mAh cm^−2^ and current density of 5 mA cm^−2^. d) Nyquist plots obtained at open‐circuit voltage over frequencies of 1 MHz to 10 mHz as a function of cycle number. e) XRD patterns along with f) photographs of the overall surface and corresponding SEM images of Zn electrodeposits after 500 cycles. g) Comparison of cycling performance in terms of cumulative capacity and cycling time with that of the following reported Zn symmetric cells featuring different separators: polyacrylonitrile (PAN),^[^ ^47^ ^]^ bacterial cellulose membrane (BCM),^[^ ^58^ ^]^ UiO‐66‐GF,^[^ ^59^ ^]^ zeolite,^[^ ^60^ ^]^ filter membrane (PM),^[^ ^61^ ^]^ Nafion,^[^ ^62^ ^]^ Janus,^[^ ^63^ ^]^ ZSM‐5,^[^ ^64^ ^]^ supramolecule‐modified glass fiber (GF@SM),^[^ ^65^ ^]^ and graphene oxide nanosheet‐modified cellulose acetate (GO/CA).^[^ ^66^ ^]^

Carbon/Zn asymmetric cell tests were performed to evaluate the reversibility of deposition/stripping during long‐term operation. The cell was operated at a high current density of 5 mA cm^−2^ with a fixed areal capacity of 1 mAh cm^−2^. The ZrOF separator exhibited 17 times higher cycling stability (7000 cycles) than that of the GF separator (400 cycles) (**Figure** **4**a). Moreover, the GF separator system exhibited a short circuit at the 400th cycle (Figure 3a, inset). As discussed above, the low ionic conductivity of Zn^2+^ ions through the GF hampered the achievement of uniform Zn^2+^‐ion mass transfer at the high current density used. The mass transfer limitation coupled with the tip effect promoted Zn dendrite growth.^[^ ^7^ ^]^ When the GF was replaced with the ZrOF, the enhanced Zn^2+^‐ion mass transfer resulted in an even Zn^2+^‐ion flux distribution across the electrode surface, promoting homogeneous Zn deposition. Consequently, the cell with the ZrOF separator operated stably for 7000 cycles without short‐circuiting. Additionally, while the average CE for the GF separator was as low as 97.9% ± 1.2% and fluctuated considerably, that of the ZrOF separator was higher (99.7% ± 0.3%), indicating a significant improvement in reversibility with each cycle. This was due to the modified coordination structure of Zn^2+^ near the negatively charged ZrOF surface. The decrease in the number of coordinated water molecules suppressed side reactions such as the HER and corrosion, resulting in a high CE close to 100%. Moreover, the ZrOF exhibited a superior rate performance with less voltage hysteresis owing to its enhanced mass transfer characteristics (Figure 4b; Figure S17 Supporting Information). The ZrOF‐separator‐based cell exhibited dendrite‐free operation at an extremely high current density of 60 mA cm^−2^, resulting in a high CE (99.3% ± 0.6) and less voltage hysteresis (29.3 mV). At the same current density, the GF separator exhibited a lower CE (97.7% ± 3.4%) and greater voltage hysteresis (35.1 mV). Consistently, an increase in reversibility at such high current densities was also observed in the Zn/Zn symmetric cell (Figure S18, Supporting information).

To examine the changes in Zn crystallinity and morphology, the cycling stability of a Zn/Zn symmetric cell was evaluated at 1 mAh cm^−2^ and 5 mA cm^−2^ (Figure 4c). While the GF separator exhibited a short circuit owing to Zn dendrite formation at 300 h, the ZrOF separator showed stable charge–discharge behavior for 3000 h. The surface deterioration of the Zn anode was studied by EIS‐based resistance analysis as a function of cycle number (Figure 4d; Figure S19, Supporting Information). The GF separator achieved an increase in the uncompensated resistance (*R*_u_) from 2.8 to 34.7 Ω cm^2^ owing to the formation of a nonconductive ZHS layer on the electrode surface, which was due to the HER that consumed H^+^ and produced OH^−^ in the vicinity of the Zn anode surface.^[^ ^57^ ^]^ This result was corroborated by the XRD patterns of the Zn electrodeposits after 500 cycles (Figure 4e). For the GF separator, the peaks at 2*θ* = 12.2° and 24.6° were assigned to the ZHS layer (see green box in Figure 4e). In contrast, for the ZrOF separator, no ZHS formation was observed and the *R*_u_ value changed minimally during cycling. This indicated that the ZrOF obstructed the water molecules approaching the Zn anode by preferentially transporting Zn^2+^ ions through surface conduction.

The GF separator exhibited a relatively low crystallinity ratio of (002)/(100) planes, causing the vertical growth of the Zn electrodeposit and facilitating dendrite formation. When the GF was replaced with the ZrOF, the crystallinity ratio of (002)/(100) planes increased from 2.1 to 3.1, favoring the parallel growth of the Zn electrodeposit. High‐resolution SEM images confirmed that the ZrOF facilitated more uniform electrodeposition of Zn and prevented agglomeration, as evidenced by the overall surface appearance of the Zn electrodeposit after 500 charging cycles (Figure 4f). The GF‐separator‐related Zn electrodeposit exhibited heterogeneity, growing in diverse sizes and orientations while clustering together. In contrast, the ZrOF separator guided the growth of the Zn layer along a single direction parallel to the electrode surface, aligning with the (002) crystal planes. Furthermore, cross‐sectional imaging revealed that the electrodeposited Zn layer partially penetrated the GF separator, creating a rough interface, in contrast to the smooth and clear interface formed with ZrOF.

The change in the crystallinity of Zn can explain the enhanced cyclability of the ZrOF separator. Moreover, the charge–discharge performance was substantiated under different experimental conditions such as 2 mAh cm^−2^ /5 mA cm^−2^ and 1 mAh cm^−2^ /20 mA cm^−2^, underlining the superior cyclability of the ZrOF separator (Figure S20, Supporting Information). Consequently, the ZrOF separator exhibited significantly enhanced cyclability (3100 h) at a high current density of 5 mA cm^−2^ compared with that of several reported Zn symmetric cells with different separators (Figure 4g).^[^ ^47^, ^58^, ^59^, ^60^, ^61^, ^62^, ^63^, ^64^, ^65^, ^66^ ^]^

### 2.5 Cell Performance of Zn–I_2_ Battery with ZrOF Separator

**Figure 5:**
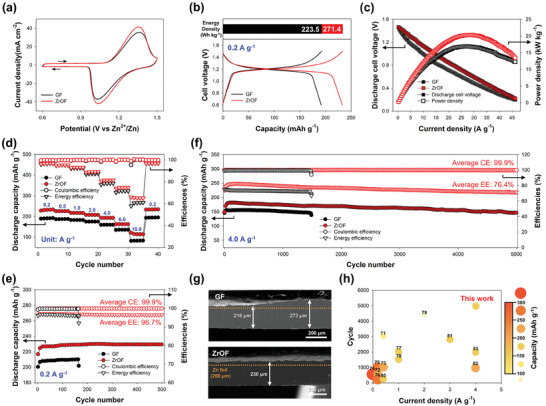
Cell performance of Zn–I_2_ batteries with GF or ZrOF as separator. a) CV curves acquired at a scan rate of 1.0 mV s^−1^. b) Galvanostatic charge–discharge voltage curves and corresponding energy densities at 0.2 A g^−1^. c) Discharge polarization and power density curves as a function of current density at 100% state of charge. d) Variation in discharge capacity and CE at various current densities from 0.2 to 1.0 A g^−1^. e) Cycling stability of Zn–I_2_ battery at a constant current density of 0.2 A g^−1^ (equivalent to 2.6 mA cm^−2^). f) Extended cycling performance test at a high current density of 4 A g^−1^ (equivalent to 51.5 mA cm^−2^). g) Cross‐sectional SEM images of Zn metal electrodes after the 1000th discharge cycle. h) Performance comparison with previously reported Zn–I_2_ batteries.

To evaluate the applicability of the ZrOF separator in a Zn–I_2_ full cell, CV tests of Zn–I_2_ batteries with the GF or ZrOF as the separator were performed at scan rates of 0.1–2 mV s^−1^ using 2.0 m ZnSO_4_ and 0.3 m ZnI_2_ as the aqueous electrolyte (**Figure** **5**a; Figure S21, Supporting Information). The cell with the ZrOF separator showed improved peak potential separation and increased peak current density, reflecting the enhanced reversibility and mass transfer characteristics of Zn^2+^ ions, respectively.

Owing to its enhanced ion mobility, the ZrOF separator enabled the Zn–I_2_ battery to exhibit remarkably improved galvanostatic charge–discharge performance at a current density of 0.2 A g^−1^, corresponding to an areal current density of 2.6 mA cm^−2^ (Figure 5b). The available capacity and energy density were extended to 233.1 mAh g^−1^ and 271.4 Wh kg^−1^, respectively, which were ≈17% higher than those of the GF separator. Additionally, the ZrOF separator exhibited a smaller average charge–discharge voltage difference (66.7 mV) than that of the GF separator (84.9 mV), indicating reduced energy losses during the electrochemical process owing to its high Zn^2+^‐ion mobility and fast redox kinetics. This energy benefit signified that the ZrOF separator delivered an excellent power density under operating conditions with high current density. The specific power density and corresponding discharge polarization curves were monitored as a function of current density (Figure 5c). The ZrOF separator exhibited a relatively low voltage drop and delivered an excellent power density of 20.3 kW kg^−1^ at 28.6 A g^−1^, whereas the GF separator showed a value of 16.9 kW kg^−1^ at 27.4 A g^−1^. The ZrOF‐separator‐based battery significantly outperforms other Zn‐based batteries in terms of specific power density.^[^ ^67^, ^68^, ^69^, ^70^ ^]^

Rate performance tests were conducted at current densities, ranging from 0.2 to 10 A g^−1^ (Figure 5d). The contrast in performance between the ZrOF and GF separators became more pronounced at elevated current rates. The ZrOF separator achieved good cell performance with an energy efficiency of 65.3% and a discharge capacity of 115.1 mAh g^−1^ at an extremely high current density of 10 A g^−1^. In contrast, the GF separator showed poor cell performance (60.2% and 82.1 mAh g^−1^) at the same current density owing to its large charge–discharge voltage polarization (Figure S22, Supporting Information). These results indicate that the ZrOF separator maintains a good balance between energy density and power density, rendering it a promising energy storage system with long‐term stability and high‐rate performance.

The long‐term stability of the Zn–I_2_ battery was evaluated at a current density of 0.2 A g^−1^ (Figure 5e). The GF separator exhibited severe performance degradation after 167 cycles, and a Zn‐dendrite‐induced short circuit was observed in the corresponding charge–discharge voltage profiles (Figure S23, Supporting Information). In contrast, the ZrOF separator demonstrated stable performance for 500 cycles with a high capacity of 229.35 mAh g^−1^ (2.95 mAh cm^−2^), corresponding to a capacity retention of 99.62%. This was related to the enhancement in the Zn anode stability caused by the suppression of the HER and corrosion. Additionally, the negatively charged ZrOF surface impeded the crossover of charged cathodic species, particularly I_3_^−^, through electrostatic repulsion. In the Zn–I_2_ battery cathode, I_2_ is in dynamic equilibrium with the highly soluble I_3_^−^, which may diffuse to the Zn anode surface, leading to self‐discharge and deteriorating stability of the Zn anode. The negatively charged ZrOF surface effectively repelled anions, preventing the diffusion of I_3_^−^ ions to the anode and thus resulting in a high capacity retention (≈100%). This is more obvious from the open circuit voltage (OCV) retention test and self‐discharge test (Figure S24, Supporting information). ZrOF gave the lower voltage decay rate during rest period and higher CE during re‐discharge after rest.

Even during cycling at a high current density of 4 A g^−1^ (corresponding to an areal current density of 51.5 mA cm^−2^), the ZrOF‐separator‐based Zn–I_2_ battery exhibited a remarkably extended cycling lifespan to 5000 cycles, maintaining an average CE of 99.9% and an average EE of 76.4% (Figure 5f). Furthermore, the capacity was maintained above 80% throughout cycling. The noteworthy cycling stability was substantiated by the charge–discharge voltage profiles and Nyquist plots obtained as a function of cycle number (Figure S25, Supporting Information). As the number of cycles increased, the polarizations and resistances for the GF separator sharply increased compared with those for the ZrOF variant. Moreover, cross‐sectional images of the Zn anode cycled 1000 times revealed that the Zn electrodeposit on the ZrOF separator was more uniform than that on the GF separator, preventing early Zn‐dendrite‐induced cell failure (Figure 5g). Crystallinity analysis corroborated the parallel growth of the Zn electrodeposit with a high ratio of (002)/(100) planes and the suppressed HER with undetectable ZHS precipitation (Figure S26, Supporting Information). Consequently, the ZrOF‐separator‐based cell outperforms several reported Zn–I_2_ batteries as it exhibits high capacity (181.6 mAh g^−1^) and cycling stability (>5000 cycles) even at a high current density (4 A g^−1^) (Figure 5h; Table S1, Supporting Information).^[^ ^71^, ^72^, ^73^, ^74^, ^75^, ^76^, ^77^, ^78^, ^79^, ^80^, ^81^, ^82^, ^83^ ^]^

## 3 Conclusion

In this study, the surface conduction of Zn^2+^ ions through a fibrous ZrO_2_ continuum was explored to enhance their mass transfer characteristics and modify their water solvation structure. Physicochemical and computational analyses were conducted to investigate the structural interactions between Zn^2+^, ZrO_2_, and H_2_O. The findings indicated that the Zn^2+^ ions with fewer solvated H_2_O molecules (≈2) were stabilized by strong Zr─O─Zn bonding. The robust interactions between Zn^2+^ ions and the ZrOF drove surface conduction to become the predominant mode of ion transport, leading to a 6.54‐fold enhancement in the single‐Zn‐ion conductivity. Notably, the contribution of surface conduction to the overall single‐Zn^2+^‐ion conduction was 53.2%. During surface conduction, the reduced solvation number helped suppress the HER at the electrode surface, as evidenced by a 58.8% decrease in hydrogen generation during cell cycling. Consequently, by adjusting the transport mechanism of Zn^2+^ ions within the bulk electrolyte regime using fibrous ZrOF, the irreversibility issues of Zn anode stemming from dendrite growth and the HER were tackled, leading to a noteworthy cyclability of 3100 h at a high current density of 5 mA cm^−2^. The enhancement in electrode irreversibility was clarified by comparing the electrode morphology and crystallinity after cycling. Furthermore, the ZrOF‐based Zn–I_2_ full cell demonstrated both high rate capability (20.3 kW kg^−1^ at 28.6 A g^−1^) and remarkable cyclability (>5000 cycles at 181.6 mAh g^−1^ and 4 A g^−1^), outclassing numerous reported Zn‐based batteries.

## Conflict of Interest

The authors declare no conflict of interest.

## Author Contributions

J.S.C., C.L., and J.H.Y. designed the work. J.S.C., Y.H., E.J.Y., and D.G. prepared the samples and carried out the physicochemical and electrochemical characterizations. S.P., J.M.Y. and C.L. performed the calculations. J.S.C, Y.H., and E.J.Y. evaluated the battery performance. J.S.C., Y.K., and Y.J.H. did the data curation and project administration. J.S.C. and J.H.Y. analyzed the experimental data. J.S.C., S.P., C.L., and J.H.Y. wrote and edited this manuscript. All authors have given approval to the final version of the manuscript.

## Supporting information



Supporting Information

## Data Availability

The data that support the findings of this study are available from the corresponding author upon reasonable request.
