# Supplementary material for: Stable Zinc Metal Battery Development: Using Fibrous Zirconia for Rapid Surface Conduction of Zinc Ions With Modified Water Solvation Structure
Source: Small. 2024 Oct 28;21(1):2406481. doi: 10.1002/smll.202406481 (PMC11707580; doi:10.1002/smll.202406481)
Supplement: Supplementary file 1 — Supporting Information [file SMLL-21-2406481-s001.docx]

Supporting Information

Stable Zinc Metal Battery Development: Using Fibrous Zirconia for Rapid Surface Conduction of Zinc Ions with Modified Water Solvation Structure

*Jin Seong Cha, Sanghyeon Park, Yuna Hwang, Eun Jeong Yoon, Donghee Gueon, Jong Min Yuk, Yun-Chan Kang, Chan-Woo Lee,* and Jung Hoon Yang**

**S1. Experimental Section**

*Materials:* All solutions were prepared with deionized (DI) water. Zinc sulfate heptahydrate (ZnSO_4_·7H_2_O, 99%) and zinc iodide (ZnI_2_, 98%) were purchased from Sigma-Aldrich. Sodium sulfate decahydrate (Na_2_SO_4_·10H_2_O, 99%) was obtained from Alfa Aesar. Unless otherwise specified, all chemicals were used without further purification. Separators, including glass fibers (GF/C) and ZrO_2_ felt (ZYF-50), were purchased from Whatman™ and Zircar, respectively. Zn foil (200-µm-thick), carbon-polymer bipolar plate (Sigracell TF6), and activated carbon cloth (ACC-5092-20) were purchased from Goodfellow, SGL BP, and Kynol, respectively.

*Material Characterization*: Zeta potential distribution was measured by dynamic light scattering (DLS; Litesizer 500, Anton Paar) using a suspension containing 0.1 wt% ZrOF. To prepare the suspension, 0.1 wt% ZrOF was dispersed in a ZnSO_4_ solution, followed by magnetic stirring and agitation in an ultrasonic bath for 10 min to ensure uniform particle dispersion. The surface and cross-sectional morphologies of the samples were examined by field-emission scanning electron microscopy (FE-SEM; S-4800, Hitachi) at an accelerating voltage of 5 kV. Samples for cross-sectional analysis were prepared using an ion milling system (E-3500, Hitachi). Energy-dispersive X-ray (EDX) spectroscopy (Unti^®^ Max, Oxford Instruments) was performed to map the elements on the separator surface at an accelerating voltage of 20 kV. The amount of Zn^2+^ ions adsorbed on the separator surface was estimated by inductively coupled plasma-optical emission spectrometry (ICP-OES) after immersing a ZrOF fragment (2.5 g) in a ZnSO_4_ solution (5 mL, 50 mm) for over 24 h. Variations in the bonding energy of each element were investigated by multipurpose X-ray photoelectron spectroscopy (XPS; Sigma Probe, Thermo Fisher). All XPS profiles were collected using a K-Alpha instrument operating with Al Kα radiation (1486.7 eV) and calibrated with the binding energy of sp^2^ carbon in the C 1*s* peak (284.6 eV). High-resolution powder X-ray diffractometry (XRD; SmartLab, Rigaku) was conducted to identify the crystalline structure of Zn in a 2*θ* range of 5°–80° at a scan rate of 5° min^−1^.

*Electrochemical Analysis*: Electrochemical measurements were conducted using a VSP electrochemical workstation (Biologic) with a custom-made three-electrode cell featuring bipolar carbon or metallic Zn substrates as the working electrode, a Zn plate as the counter electrode, and Ag/AgCl (3 m NaCl) as the reference electrode, with 2 m ZnSO_4­_ as the electrolyte.^[S1]^ The GF- or ZrOF-based separator was tightly secured in front of the working electrode, whose apparent area was 0.196 cm^2^. CV data were recorded at a scan rate of 20 mV s^−1^ over a potential range of −0.6 to −1.1 V. CA curves were acquired at a constant overpotential of 400 mV for 600 s. Tafel polarization curves were obtained at a scan rate of 10 mV s^−1^ within an overpotential range of ±150 mV. Using 1 m Na_2_SO_4_ as the electrolyte, linear sweep voltammetry (LSV) data were collected at a scan rate of 10 mV s^−1^ in a potential range of −1.2 V to −2.0 V.

To quantitatively measure hydrogen evolution, the internal pressure of the Zn symmetric cell was monitored as a function of cumulative cycles. The cutoff capacity and current density were set to 1 mAh cm^−2^ and 1 mA cm^−2^, respectively. Real-time pressure data were recorded at 0–2 kPa using a low-pressure gauge (P421 series, Wise).

Sequential electrochemical impedance spectroscopy (EIS) measurements were performed as a function of cycle number for the Zn symmetric cell and Zn–I_2_ full cell. The AC voltage amplitude was set to 10 mV within a frequency range of 1 MHz to 1 mHz under the open-circuit voltage (OCV) condition.

The ionic conductivity *σ* (S cm^−1^) was determined as follows by EIS analysis using a stainless steel (SS) symmetric cell and 2 m ZnSO_4_ as the electrolyte:^[S2]^

$\sigma=\frac{L}{A\times R_{b}}$, (1)

where *L* is the thickness of the separator, *A* is the contact area, and *R*_b_ is the bulk resistance obtained from the Nyquist plot.

The Zn^2+^-ion transference number $t_{{Zn}^{2+}}$ was evaluated by measuring the liquid junction potential *E_j_* (mV) using an H-type electrolytic cell. The cell was assembled by sandwiching separators between two solution chambers filled with 0.01 m ZnSO_4_ and 0.1 m ZnSO_4_ solutions, respectively. The Ag/AgCl reference electrode was placed in each solution chamber, and the solutions were continuously stirred thereafter with a magnetic stirring bar. Subsequently, the OCV was monitored to identify the plateau region, which corresponds to the liquid junction potential. Transference numbers were calculated using Equation (2) and (3) (see Section S2 for calculation details):^[S3]^

$E_{j}=\phi^{\beta}-\phi^{\alpha}=\frac{-RT}{F}\sum_{i} \int_{\alpha}^{\beta} \frac{t_{i}}{z_{i}}d\ln a_{i}$, (2)

$\sum_{i} t_{i}=1$, (3)

where *R*, *T*, and *F* are the ideal gas constant, temperature, and Faraday constant, respectively. *ϕ* is the potential of the cell, *t_i_* is the ion transference number, *z_i_* represents the ion charge, *a_i_* is the activity of the solutions, and α and β represent phases of the two solutions. Additionally, the single-Zn^2+^-ion conductivity was obtained by multiplying *σ* and $t_{{Zn}^{2+}}$. The quantification of the ratio between surface Zn^2+^ conductivity ($\sigma_{{Zn}^{2+}}^{s}$) and bulk Zn^2+^ conductivity ($\sigma_{{Zn}^{2+}}^{b}$) for a system with the ZrOF separator is comprehensively explained in Section S3.

The activation energy for the charge-transfer reaction (*E_a_*, kJ mol^−1^) was evaluated using the Zn symmetric cell placed in a thermo-hygrostat (TC-KE-65, Jeiotech) to prevent water evaporation. *E*_a_ was determined by measuring the resistances in a temperature range of 10–60 °C and then calculated using the Arrhenius equation (Equation 4).^[S4]^

$\frac{1}{R_{ct}}=Aexp\left( -\frac{E_{a}}{RT} \right)$, (4)

where *A* is the Arrhenius slope and *R*_ct_ is the charge-transfer resistance.

The diffusion coefficient (*D*, cm^2^ s^−1^) was measured by CV analysis using 20 mm ZnSO_4_ as the electrolyte in a scan rate range of 2.5–15 mV s^−1^. Subsequently, *D* was calculated using the Randles–Ševčík equation^[S5]^

$i_{p}=2.69\times{10}^{5}n^{3/2}AC\sqrt{Dv}$, (5)

where *i_p_* is the peak current density, *n* is the number of electrons transferred in the redox reaction, *A* is the electrode area, *C* is the electrolyte concentration, and *v* is the scan rate.

*Battery Test*s: Coin cells (CR-2032) were assembled by sandwiching separators soaked with the electrolyte between electrodes under ambient conditions. Prior to use, all components were washed with ethanol and deionized water using an ultrasonic cleaner. The effective area of electrodes was 1.54 cm^2^. For the Zn/Zn symmetric cell, 200-μm-thick Zn foil was used as both the anode and cathode. In the carbon/Zn asymmetric cell, a carbon–polymer bipolar plate and Zn foil were used as the anode and cathode, respectively. In the Zn–I_2_ full cell, the ACC was used as the cathode after undergoing thermal treatment in air at 105 °C for 1 h to remove impurities. The areal loading of pure ACC was estimated to be 12.87 mg cm^−2^. Prior to use, the ACC was sufficiently wetted with the electrolyte. The electrolyte for the Zn–I_2_ battery was prepared by dissolving 2 m ZnSO_4_ and 0.3 m ZnI_2_ in DI water, and the product was strained through a syringe filter with a pore size of 0.22 µm to remove impurities. Considering that the solubility of ZnI₂ is approximately 0.476 M, the concentration was set to 0.3 M to ensure stable operation without the risk of precipitation. To assess the performance of the ZrOF as a separator in the Zn–I_2_ system, CV tests were conducted at scan rates of 0.1–2 mV s^−1^. The Zn–I_2_ full cells were tested in galvanostatic mode in a voltage range of 0.6–1.5 V using a battery test system (WBCS3000Lx32, WonATech) and then placed in an incubator chamber (IL-11-2C, Jeiotech) to maintain a constant temperature of 27.5 °C. The discharge cell voltage was recorded as a function of current density with 100% state of charge to monitor changes in power density. Rate performance tests were conducted at current densities of 0.2–10 A g^−1^. Long-term cycling tests were performed at current densities of 0.2 and 4.0 A g^−1^.

*Computational Details*: Using the Vienna Ab initio Simulation Package (VASP),^[S6]^ density functional theory (DFT) calculations and ab initio molecular dynamics (AIMD) simulations were conducted with the projector-augmented wave (PAW) scheme and spin polarization, following the Perdew–Burke–Ernzerhof (PBE) formulation.^[S7]^ A plane-wave expansion of wave functions was employed, with the cutoff energy set to 500 eV. For k-point sampling, a Monkhorst–Pack method^[S8]^ was utilized with a 1 × 1 × 1 grid. Convergence criteria for electronic and ionic steps in the DFT calculations were set to 1.0 × 10^−6^ eV and −0.02 eV Å^−1^, respectively. The Zn^2+^ ion was modeled by adjusting the total number of electrons in each system. To describe the ZrOF and bulk solution systems, a ZrO_2_ (111) slab model with cell dimensions of 14.47×14.47×27.38 Å^3^ (𝛾 = 120°; 20-Å-sized vacuum region) and a 10×10×10 Å^3^ cubic cell were constructed respectively. The adsorption energy of Zn^2+^ ions on ZrO_2_ (111) was calculated as follows:

$E_{\mathrm{Adsorption}}= E_{{\mathrm{Zn}^{2+}+ZrO}_{2}\left( 111 \right)}-E_{\mathrm{ZrO}_{2}\left( 111 \right)}-E_{\mathrm{Zn}^{2+}}$, (6)

where *E* is the DFT-derived energy.

Charge density difference maps (Figure 3d, e) were obtained using the following equations, in which *ρ* is the charge density:

$\rho_{{\mathrm{Zn}^{2+}+ZrO}_{2} (111)}- \rho_{\mathrm{ZrO}_{2} (111)}-\rho_{\mathrm{Zn}^{2+}}$ (7)

$\rho_{{\mathrm{Zn}^{2+}+ZrO}_{2} \left( 111 \right)+H_{2}O}- \rho_{{\mathrm{Zn}^{2+}+ZrO}_{2} \left( 111 \right)}-\rho_{H_{2}O}$ (8)

$\rho_{{\mathrm{Zn}^{2+}+H}_{2}O}- \rho_{\mathrm{Zn}^{2+}}-\rho_{H_{2}O}$ (9)

Bader charge analysis was performed to quantify the amount of charge transferred when the Zn^2+^ ion was stabilized by ZrO_2_ (111) and H_2_O.^[S9–11]^ AIMD simulations were conducted using the canonical NVT ensemble at 300 K using Nosé–Hoover thermostats^[S12]^ with a time step of 1 fs. Ambient H_2_O environments were modeled by placing 48 H_2_O molecules in each cell for the AIMD simulations (Figure S27). Snapshot structures obtained from 0.5 to 2.5 ps were considered when measuring their distribution.

**S2. Measurement of Zn^2+^-Ion Transference Number (**$\boldsymbol{t}_{\boldsymbol{Zn}^{\mathbf{2+}}}$**) Using Liquid Junction Potential**

The liquid junction potential *E_j_* manifests when two electrolyte solutions with disparate concentrations interface with each other. The discrepancy in the transference rates of anions and cations creates an electrically charged double layer at the junction of the solutions, enabling the formation of the liquid junction potential. The magnitude of this potential varies according to the following relationship, depending on the transference numbers of cations and anions (*t*_+_ and *t*_−_):

$E_{j}=\phi^{0.1M}-\phi^{0.01M}=\frac{-RT}{F}\sum_{i} \int_{0.01M}^{0.1M} \frac{t_{i}}{z_{i}}d\ln a_{i}=(t_{+}-t_{-})\frac{RT}{zF}\ln\frac{a_{0.01M}}{a_{0.1M}}$, (10)

where *ϕ*^0.01M^ and *ϕ*^0.1M^ represent the electromotive forces for cells with 0.01 m and 0.1 m ZnSO_4_ solutions, respectively; *a*_0.01M_ and *a*_0.1M_ represent the activity of ZnSO_4_ in the 0.01 m and 0.1 m ZnSO_4_ electrolytes, which correspond to the activity coefficients of 0.676 and 0.4, respectively;^[S6]^ *R*, *T*, and *F* are the ideal gas constant, temperature, and Faraday constant, respectively; *t_i_* is the ion transference number; and *z_i_* is the ion charge. Because the sum of the transference numbers of all participating ions is 1, that is,

$t_{+}+t_{-}=1$, (11)

the transference numbers were calculated by combining Equation (10) and (11).

The experimental measurements yielded *E_j_* values of 3.13 and −5.45 mV for GF and ZrOF, respectively. By substituting these values into the aforementioned equations, the Zn^2+^-ion transference number ($t_{{Zn}^{2+}}$) values for GF and ZrOF were obtained as 0.43 and 0.62, respectively.

**S3. Quantification of Surface Zn^2+^ Conductivity (**$\boldsymbol{\sigma}_{\boldsymbol{Zn}^{\boldsymbol{2+}}}^{\boldsymbol{s}}$**) and Bulk Zn^2+^ Conductivity** ($\sigma_{{Zn}^{2+}}^{b}$)

For GF, the total conductivity $\sigma^{t}$ encompasses the bulk Zn^2+^ conductivity ($\sigma_{{Zn}^{2+}}^{b}$) and bulk SO_4_^2−^ conductivity ($\sigma_{{SO}_{4}^{2-}}^{b}$), as follows:

$\sigma^{t}=\sigma_{{Zn}^{2+}}^{b}+\sigma_{{SO}_{4}^{2-}}^{b}$

$\sigma_{{Zn}^{2+}}^{b}:\sigma_{{SO}_{4}^{2-}}^{b}=0.43:0.57$

$\sigma_{{Zn}^{2+}}^{b}=0.754\sigma_{{SO}_{4}^{2-}}^{b}$

For ZrOF, $\sigma^{t}$ is determined considering the contribution of the Zn^2+^-ion surface conductivity ($\sigma_{{Zn}^{2+}}^{s}$) in addition to the bulk ion conductivity, which comprises both $\sigma_{{Zn}^{2+}}^{b}$ and $\sigma_{{SO}_{4}^{2-}}^{b}$. The experimentally measured Zn-ion transference number (0.62) is the sum of $\sigma_{{Zn}^{2+}}^{s}$ and $\sigma_{{Zn}^{2+}}^{b}$ relative to $\sigma^{t}$. Furthermore, assuming that the ratio of $\sigma_{{Zn}^{2+}}^{b}$ to $\sigma_{{SO}_{4}^{2-}}^{b}$ for the bulk electrolyte surrounding the ZrOF is identical to that measured for the GF, the following relationships can be derived:

For ZrOF, $\sigma^{t}={(\sigma}_{{Zn}^{2+}}^{s}+\sigma_{{Zn}^{2+}}^{b})+\sigma_{{SO}_{4}^{2-}}^{b}$

$\sigma_{{Zn}^{2+}}^{s}+\sigma_{{Zn}^{2+}}^{b}=t_{{Zn}^{2+}}\sigma^{t}=0.62\sigma^{t}$

$\sigma_{{SO}_{4}^{2-}}^{b}=\sigma^{t}-0.62\sigma^{t}=0.38\sigma^{t}$

$\sigma_{{Zn}^{2+}}^{b}=0.754\sigma_{{SO}_{4}^{2-}}^{b}=0.754\times0.38\sigma^{t}=0.29\sigma^{t}$

$\sigma_{{Zn}^{2+}}^{s}=0.62\sigma^{t}-0.29\sigma^{t}=0.33\sigma^{t}$

Ratio of surface Zn^2+^ conductivity$=\frac{0.33\sigma^{t}}{0.62\sigma^{t}}\times100\%=53.2\%$

Therefore, the contributions of the Zn^2+^-ion surface conductivity to the total ion conductivity and single-Zn^2+^-ion conductivity were 33% and 53.2%, respectively.

**
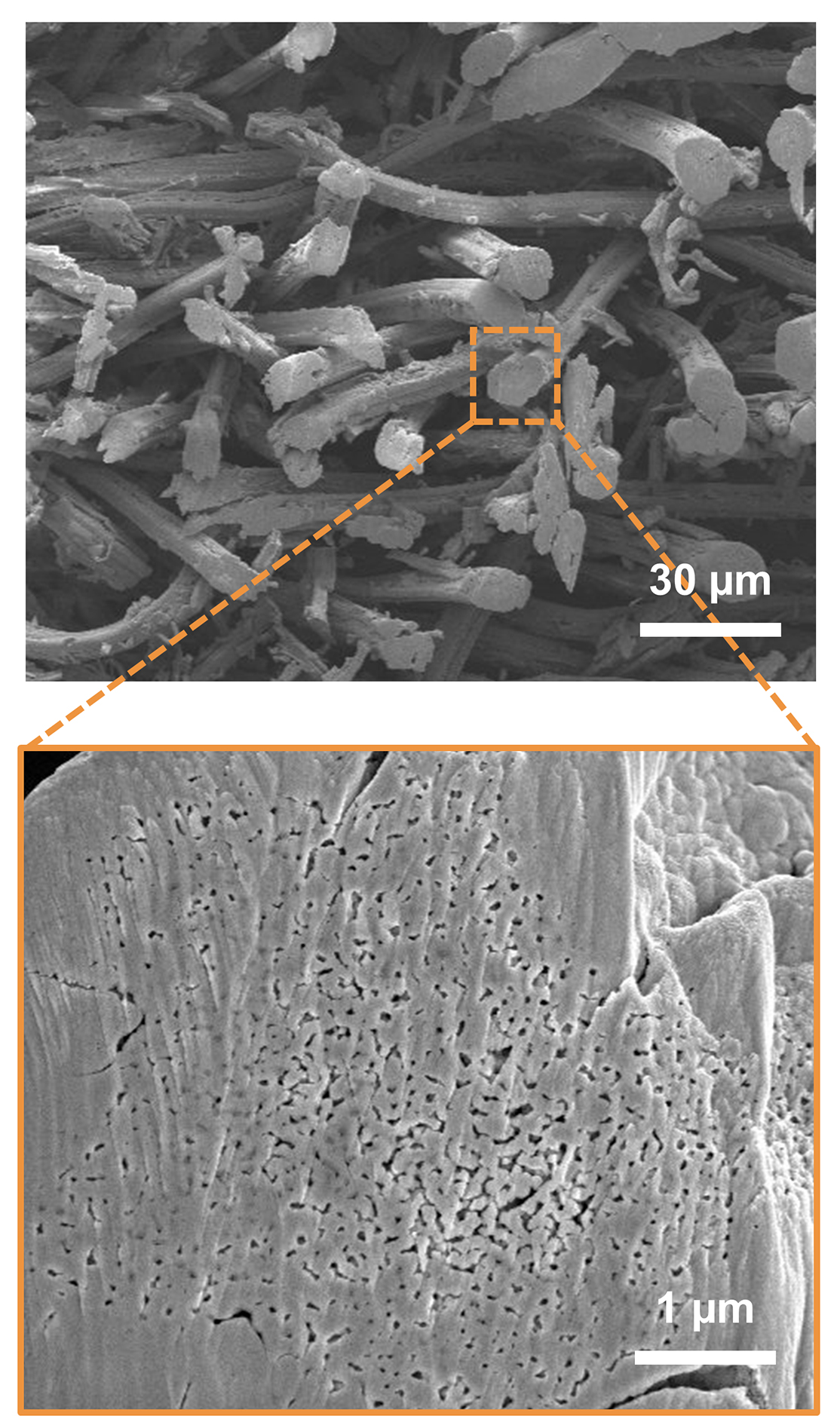
**

**Figure S1.** SEM images of cross-section of ion-milled ZrOF.


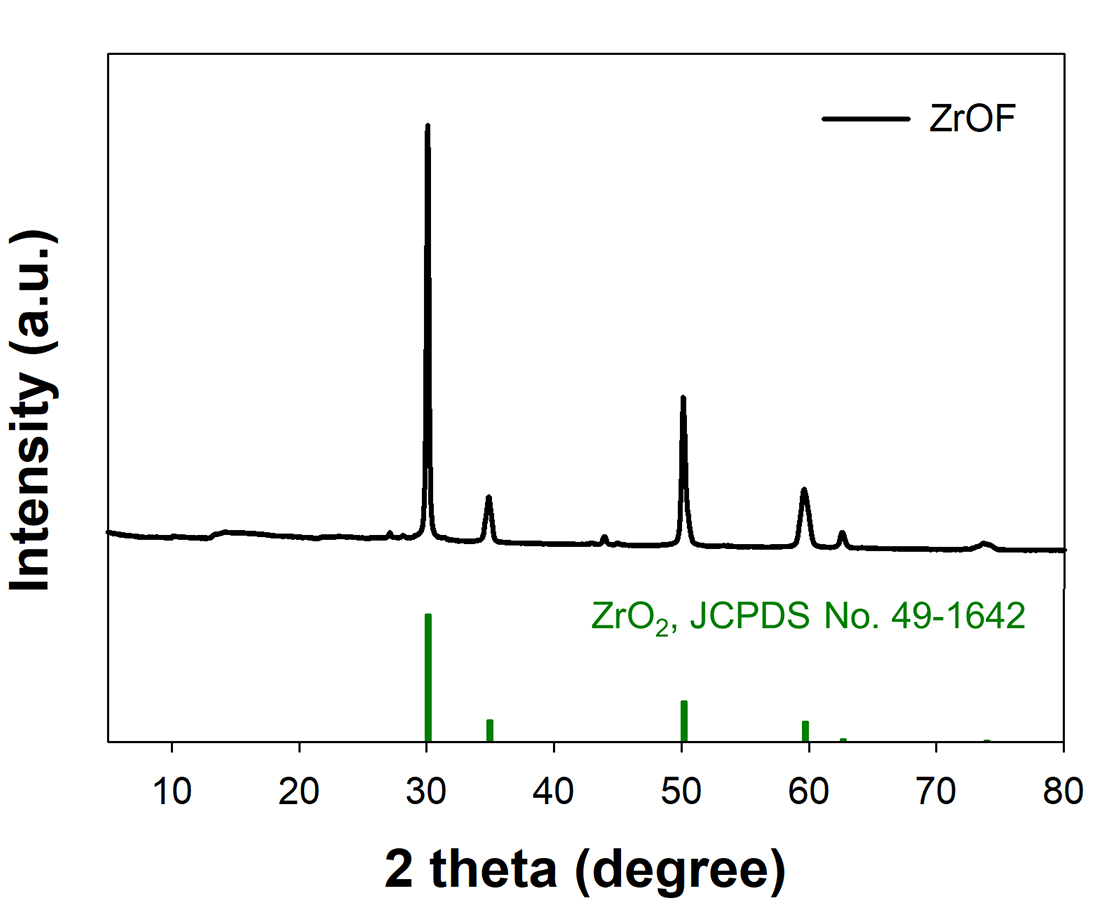


**Figure S2.** XRD pattern of ZrOF.


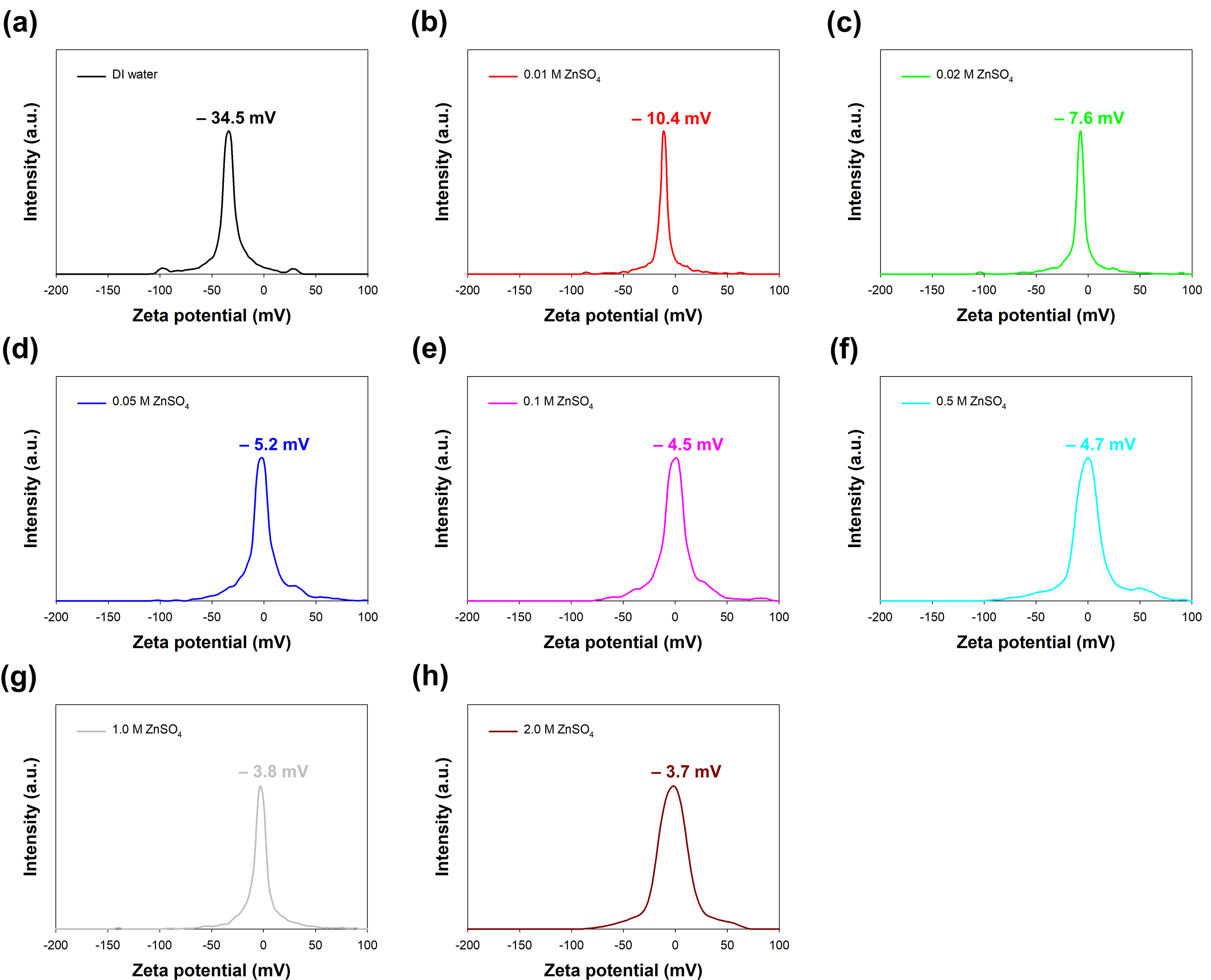


**Figure S3.** Zeta potential distribution curves of ZrOF powders dispersed in ZnSO_4_ solutions with molar concentrations of a) 0, b) 0.01, c) 0.02, d) 0.05, e) 0.1, f) 0.5, g) 1.0, and h) 2.0 m (mol L^−1^).


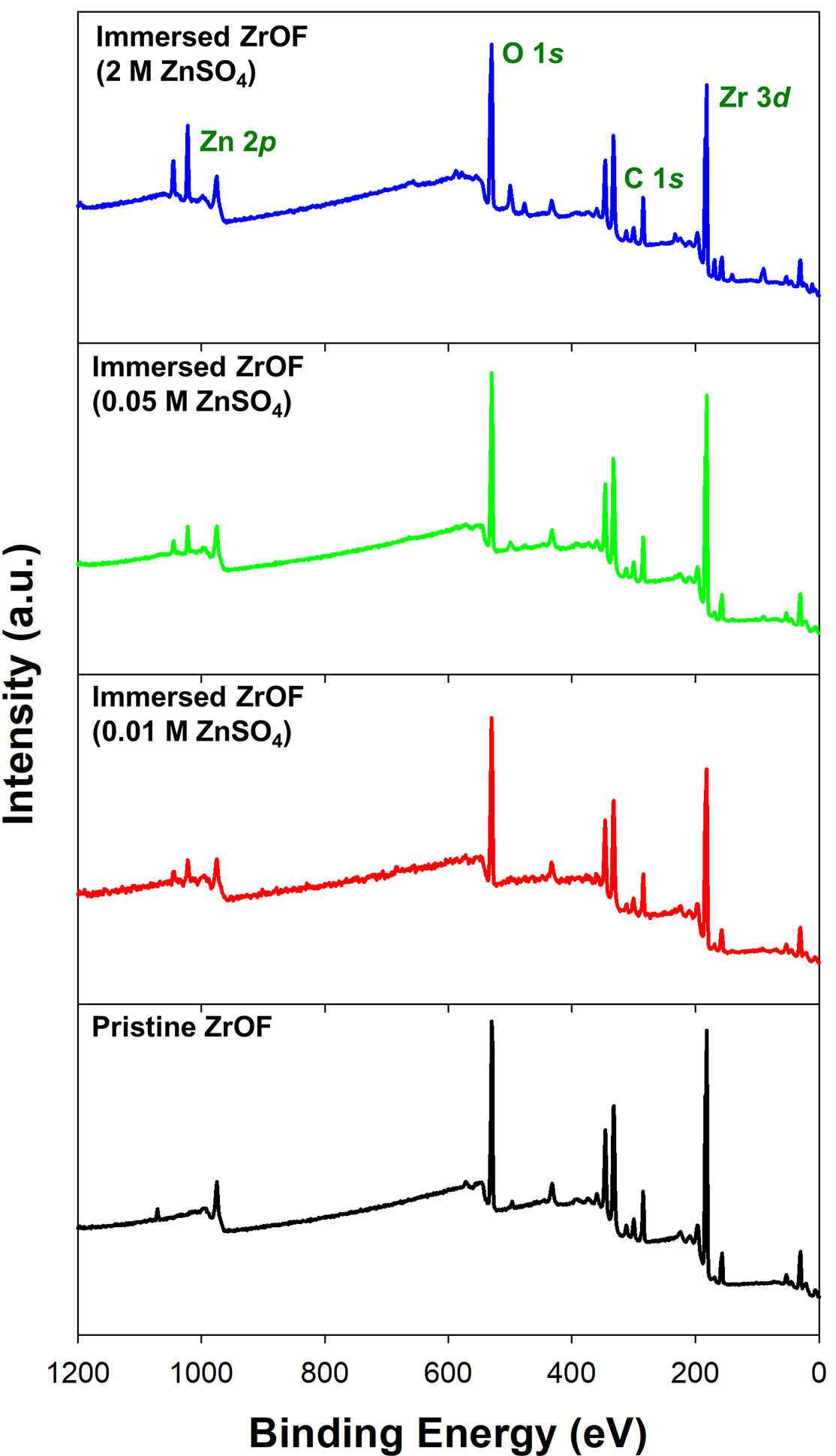


**Figure S4.** Overall XPS survey spectra of pristine and immersed ZrOF specimens.


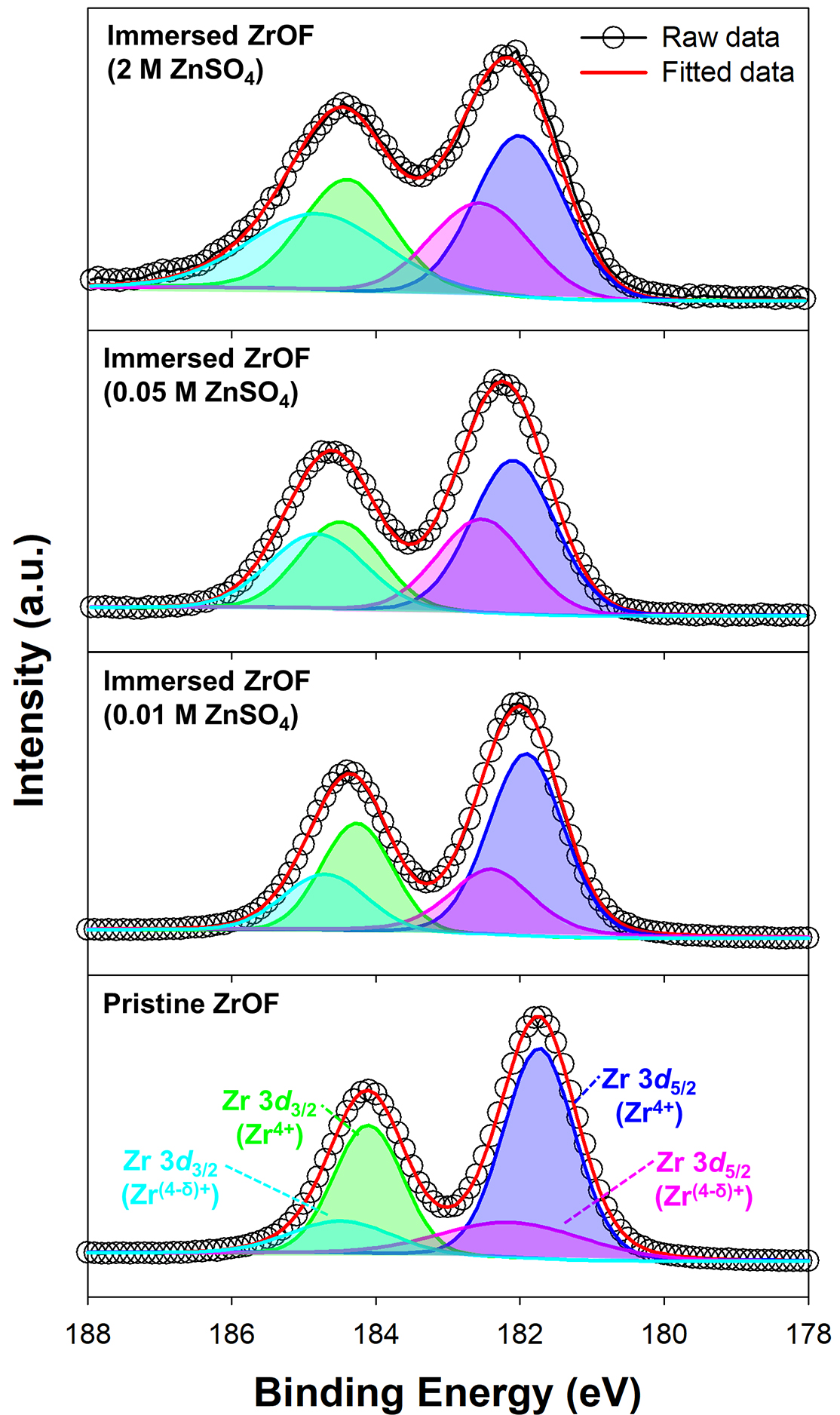


**Figure S5.** High-resolution Zr 3*d* spectra of pristine ZrOF and ZrOF immersed in *x* m ZnSO_4_ solutions (*x* = 0.01, 0.05, and 2).


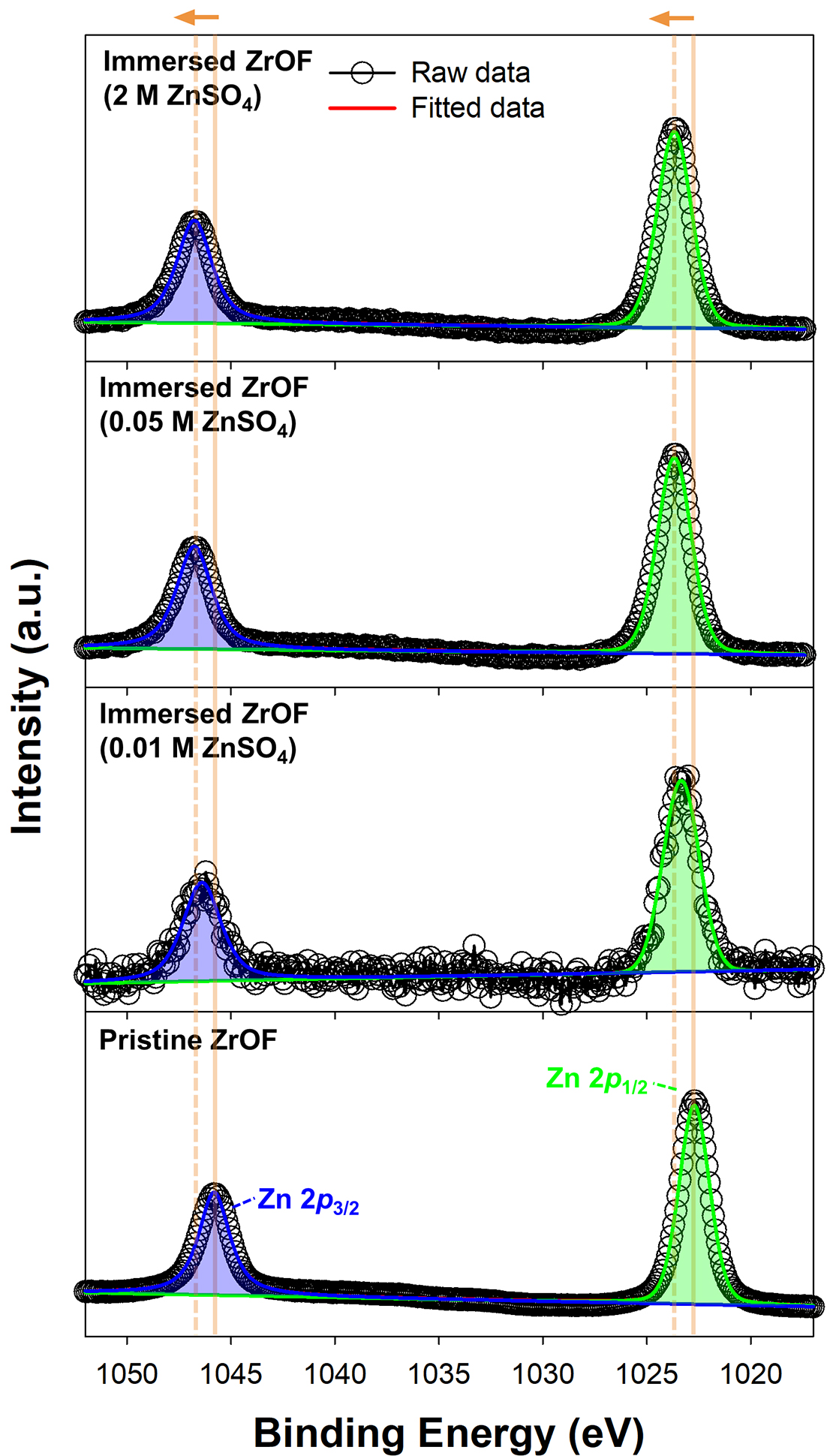


**Figure S6.** High-resolution Zn 2*p* spectra of pristine ZrOF and ZrOF immersed in *x* m ZnSO_4_ solutions (*x* = 0.01, 0.05, and 2).


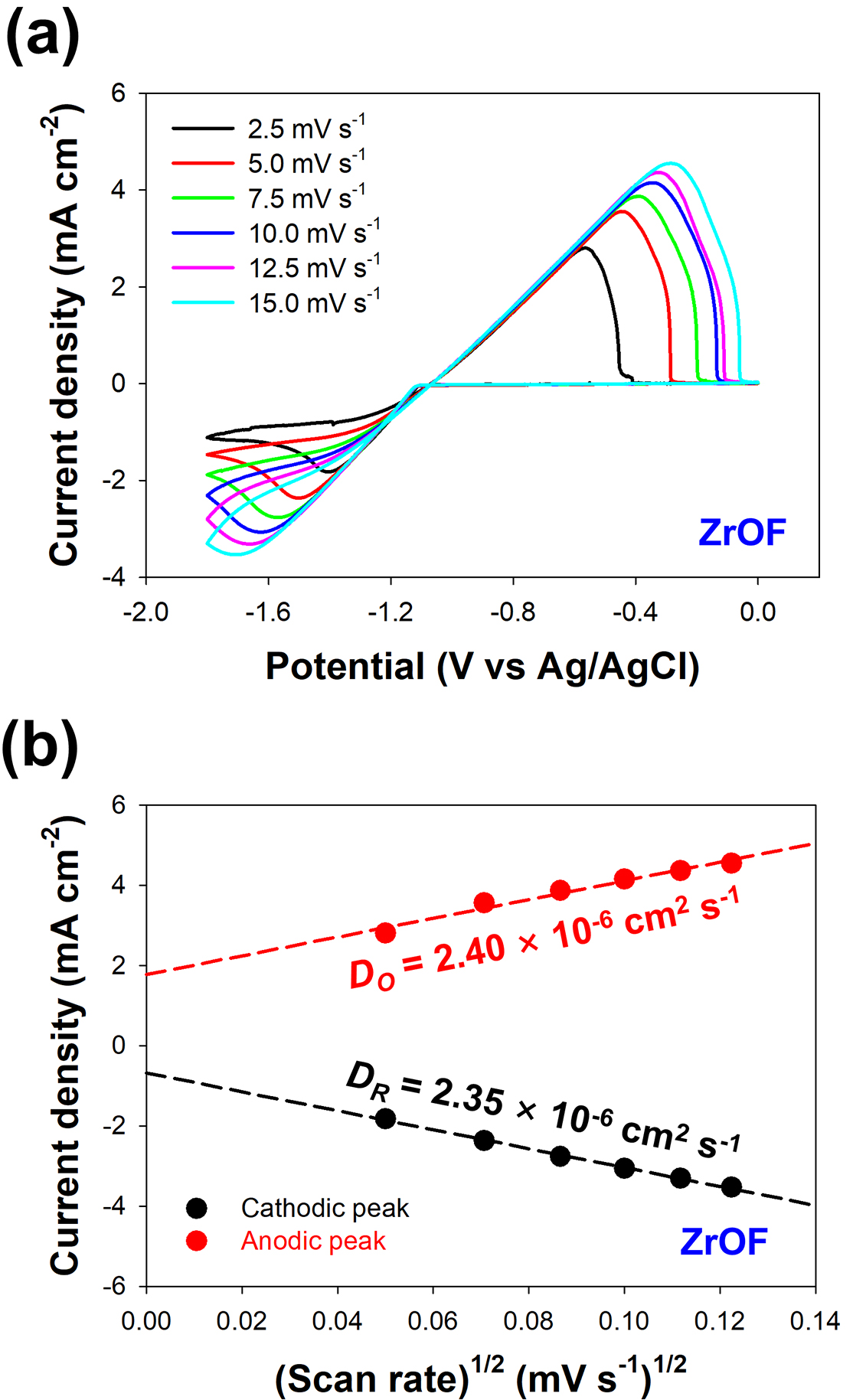


**Figure S7.** a) CV curves of ZrOF separator obtained at scan rates of 2.5–15 mV s ^−1^ using a 0.02 m ZnSO_4_ solution with a three-electrode system (working/counter electrodes: SGL carbon plate; reference electrode: Ag/AgCl). b) Diffusion coefficients calculated using the Randles–Ševčík equation.


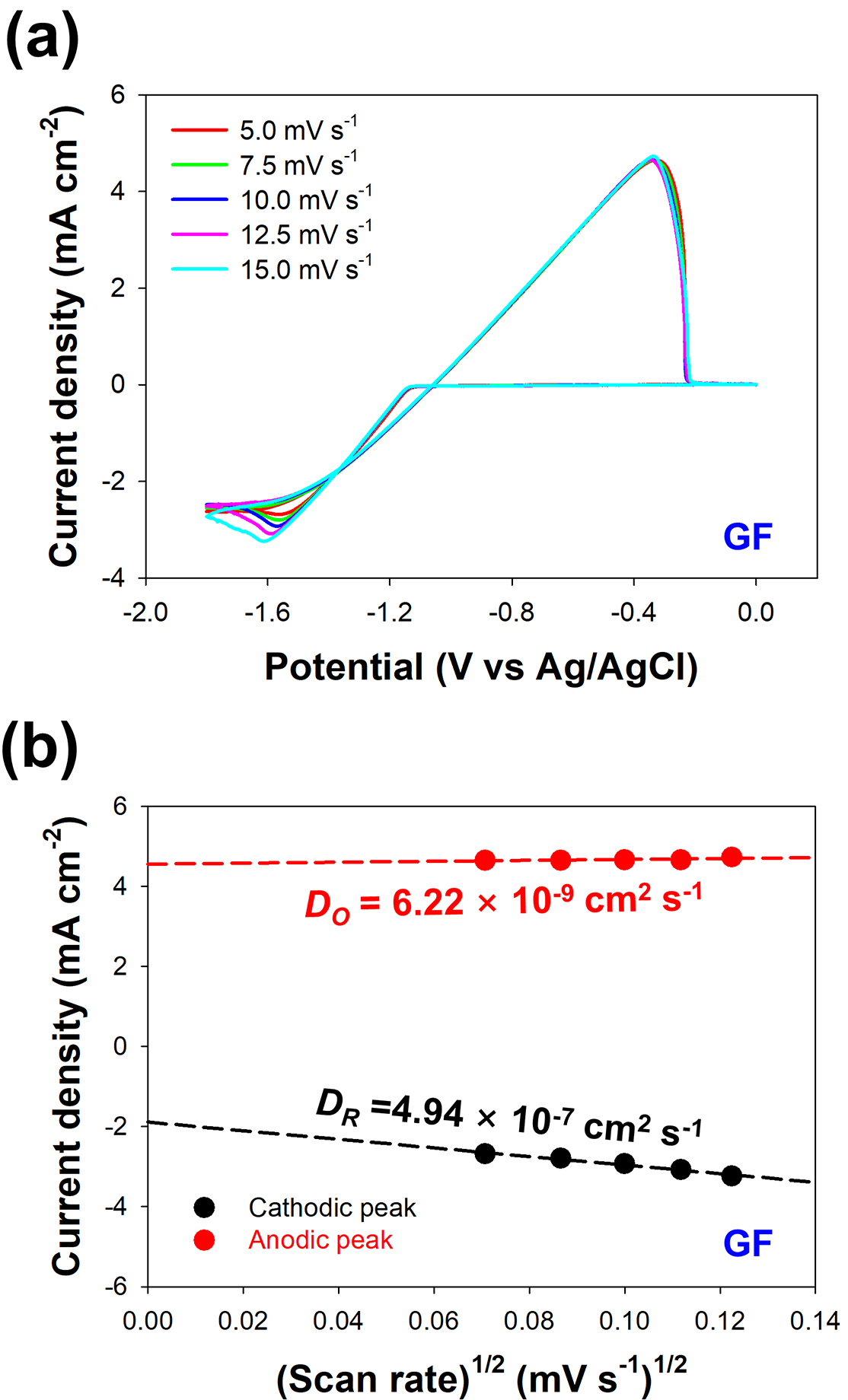


**Figure S8.** a) CV curves of GF separator obtained at scan rates of 5.0–15 mV s ^−1^ using a 0.02 m ZnSO_4_ solution with a three-electrode system (working/counter electrodes: SGL carbon plate; reference electrode: Ag/AgCl). b) Diffusion coefficients calculated using the Randles–Ševčík equation.


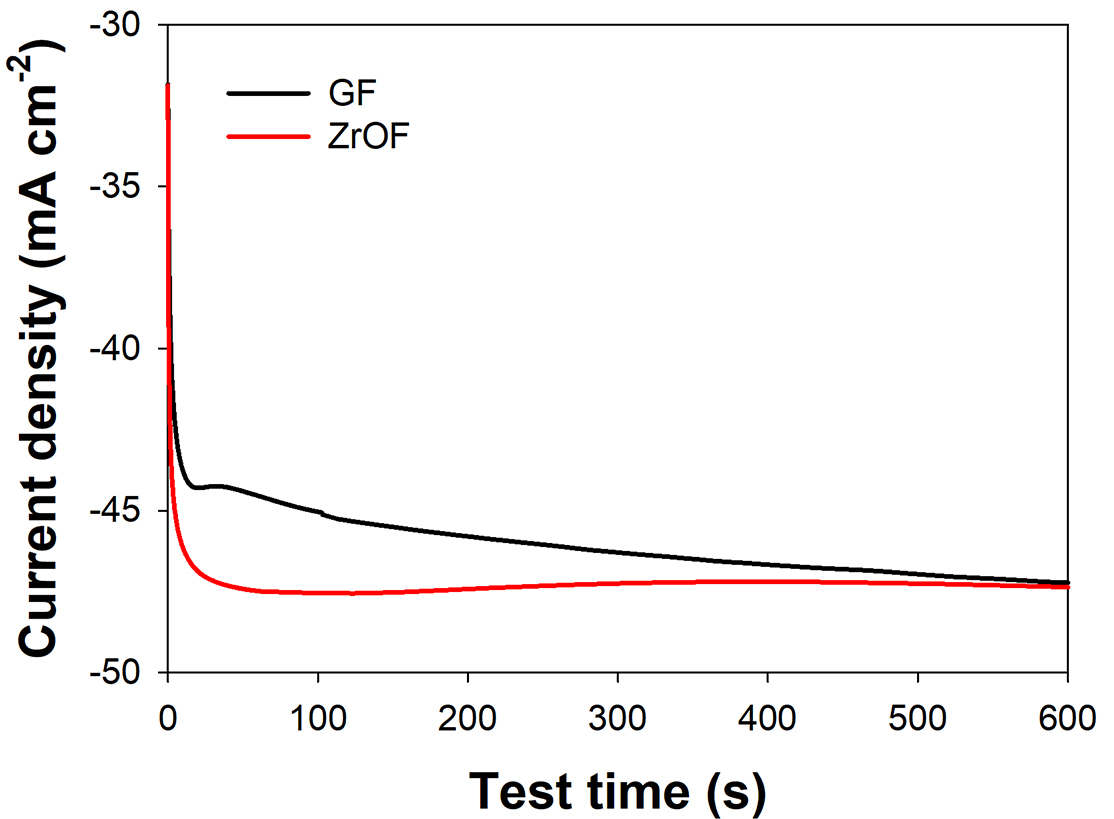


**Figure S9.** CA curves obtained at a constant overpotential of 400 mV for 600 s.


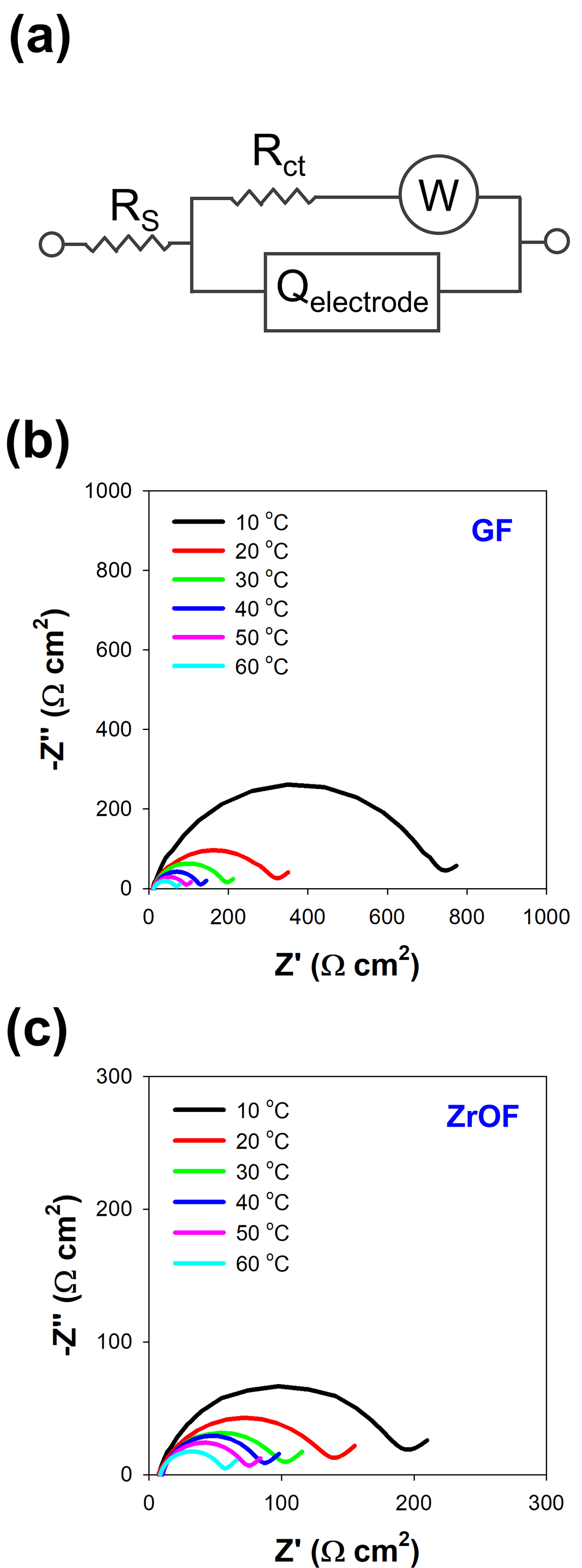


**Figure S10.** a) Equivalent circuit model for EIS analysis. Nyquist plots acquired at different temperatures (10–60 °C) for b) GF and c) ZrOF separators.

**
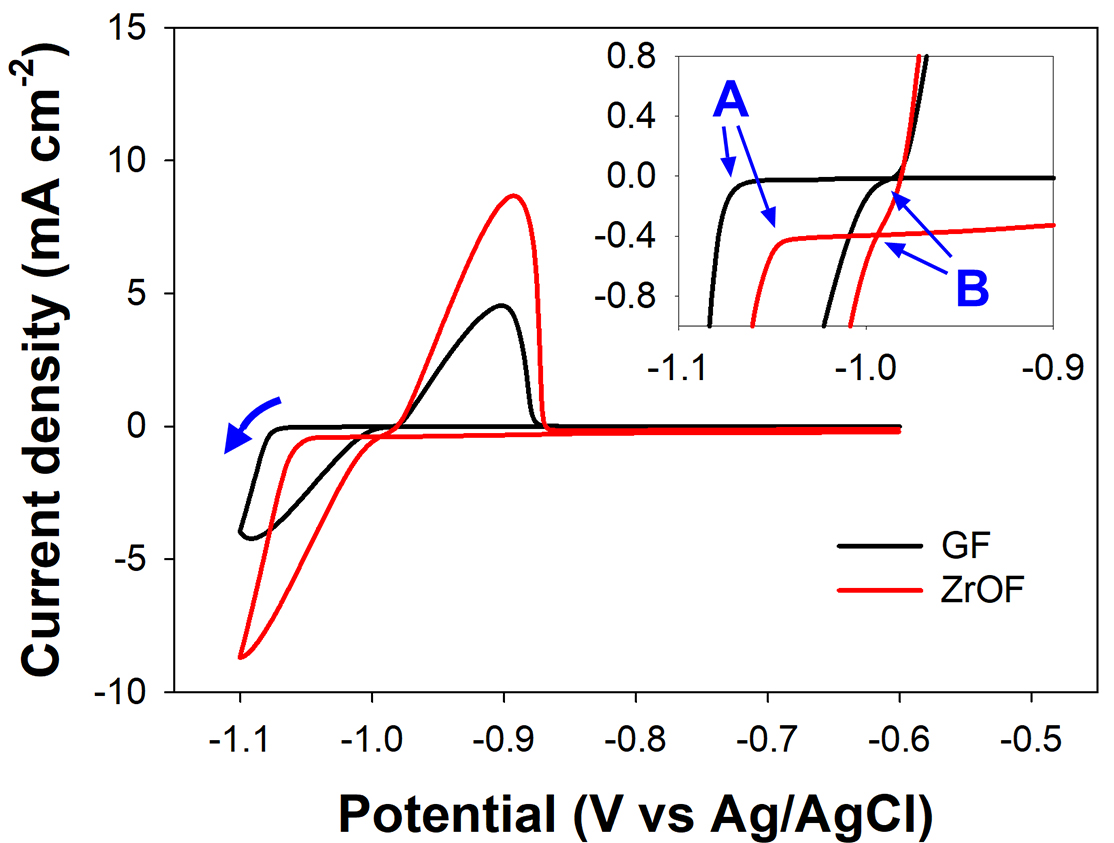
**

**Figure S11.** CV curves for Zn^0^/Zn^2+^ redox reaction at a scan rate of 20 mV s^−1^.


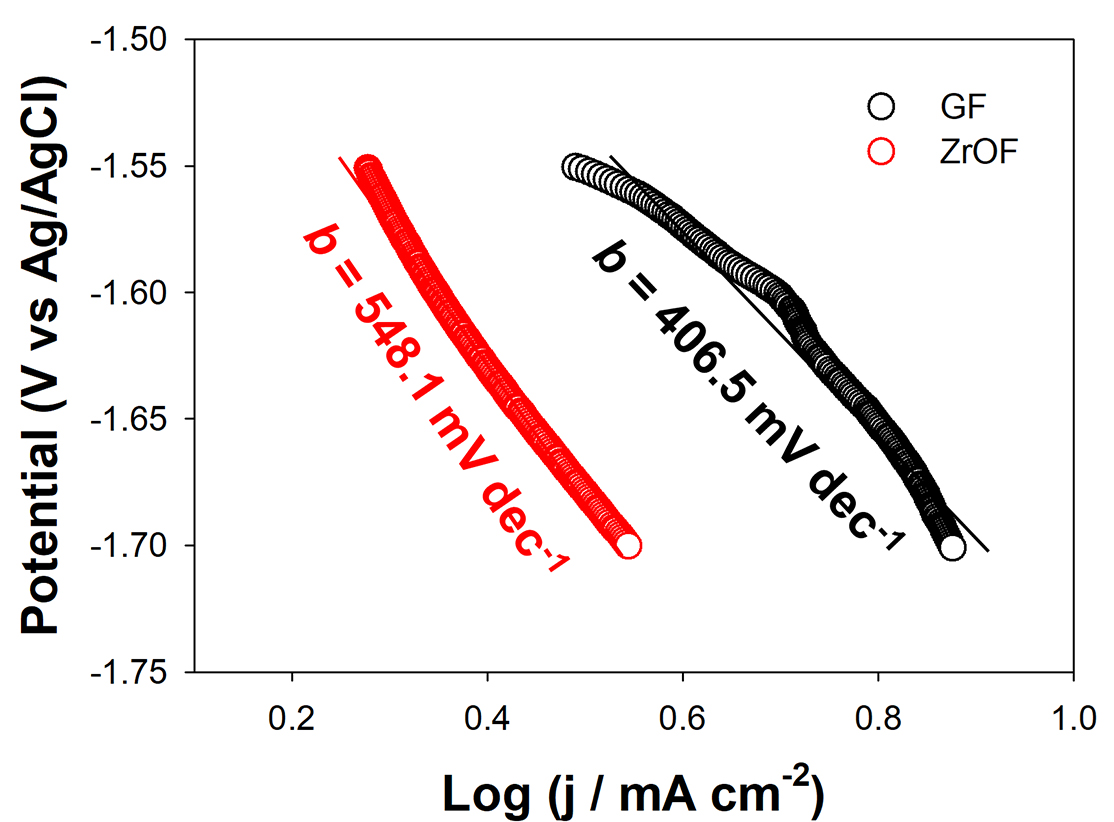


**Figure S12.** Tafel polarization curves reconstructed from LSV data shown in Figure 2e.

**
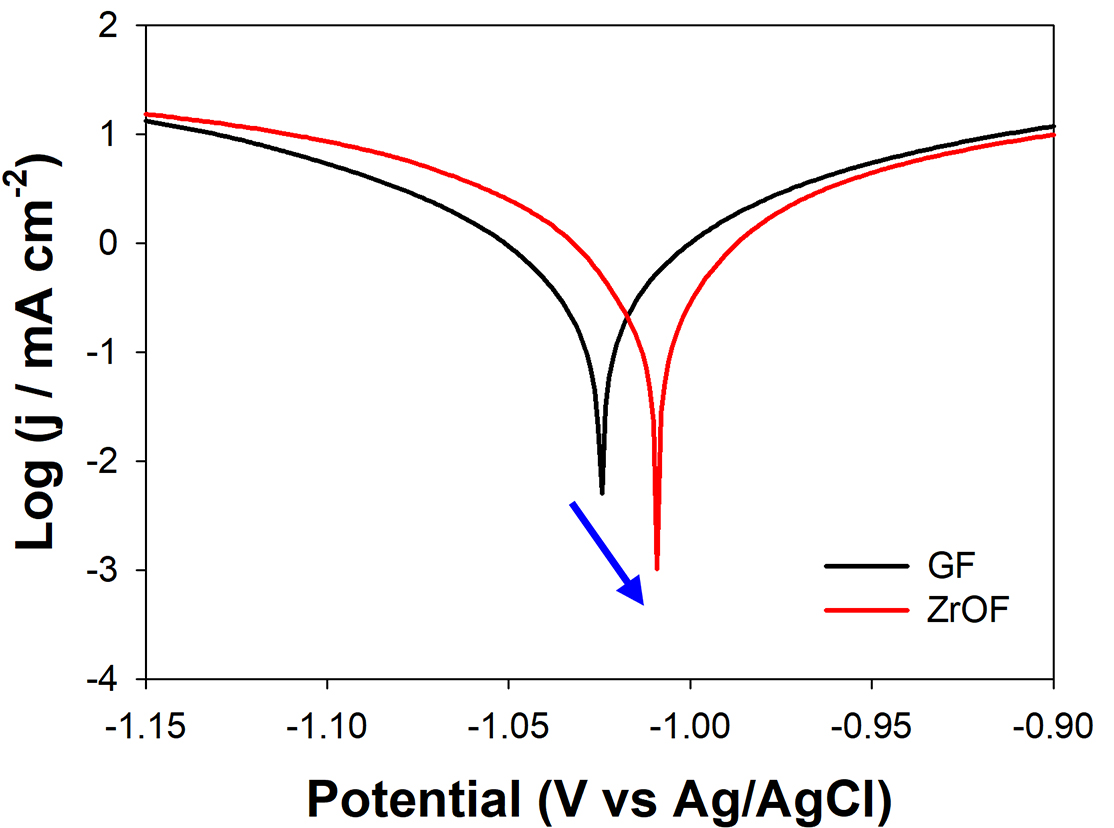
**

**Figure S13.** Tafel polarization curves for corrosion at a scan rate of 10 mV s^−1^.

**
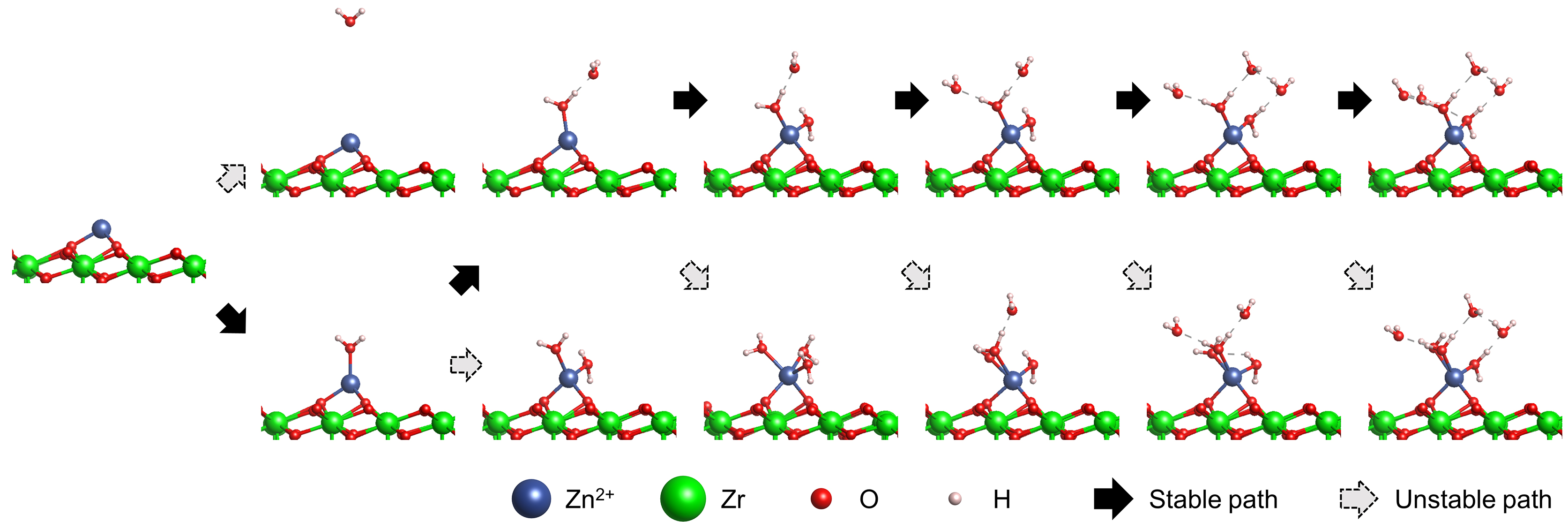
**

**Figure S14.** Hydration of Zn^2+^ ion on ZrO_2_ (111) surface.


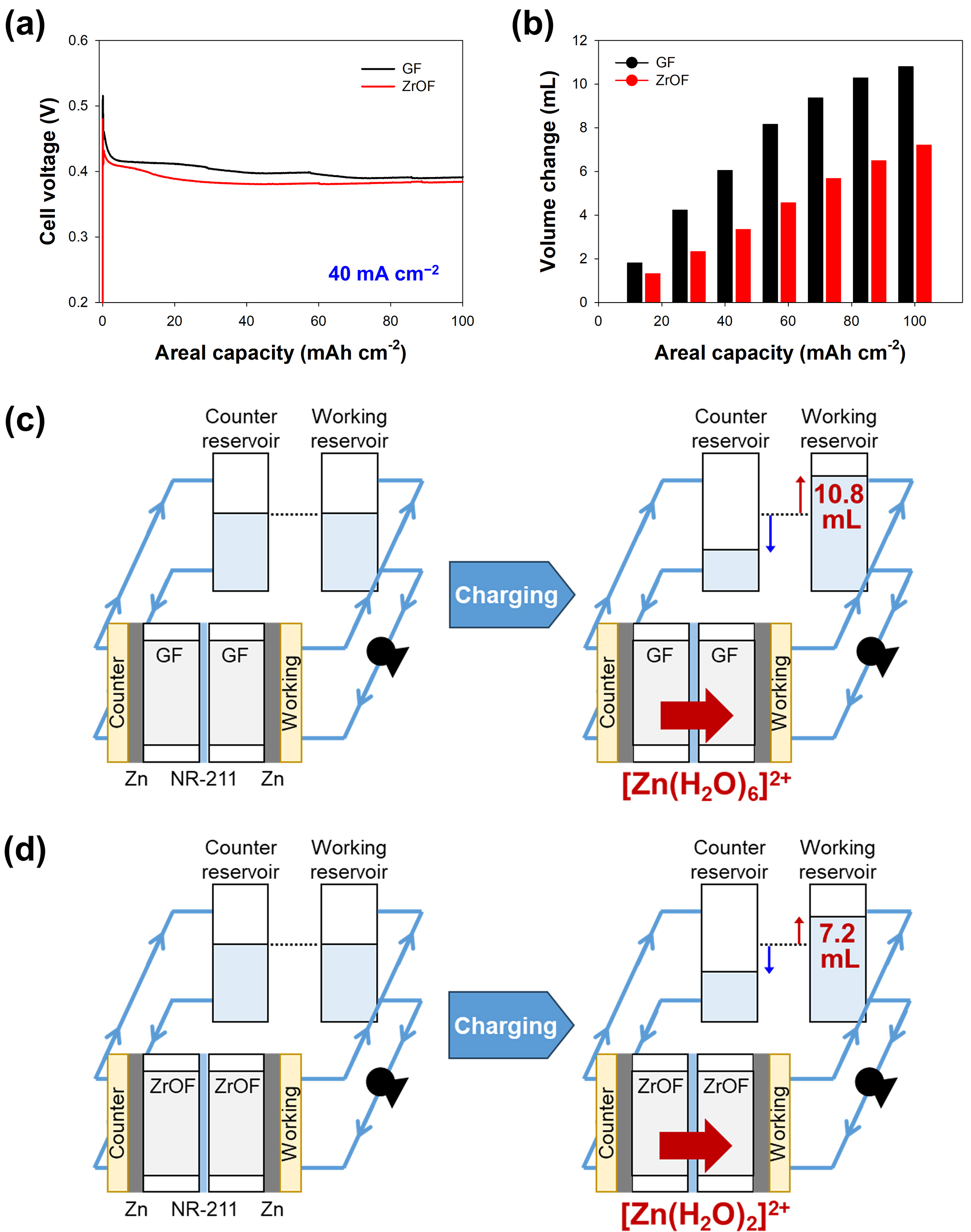


**Figure S15**. a) Voltage profiles during galvanostatic Zn deposition at 40 mA cm^−2^. b) The volume change of the electrolyte in the anode and cathode due to Zn^2+^ migration during the charging test. Schematic diagram of Zn//Zn symmetric flow cell and changes in reservoir volume for c) GF separator and d) ZrOF separator.


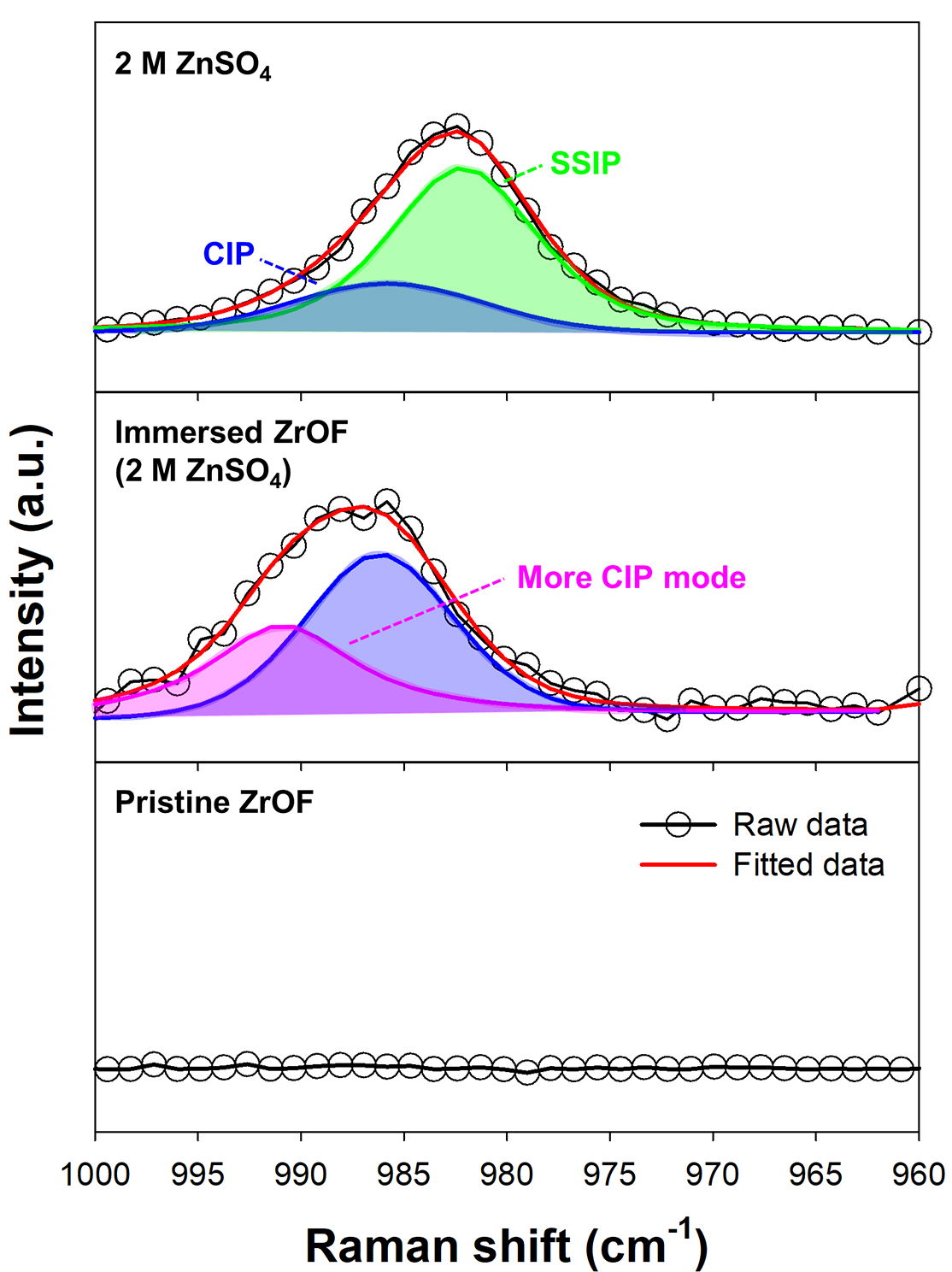


**Figure S16**. Raman spectra for *v*(SO_4_^2−^) band at 960– 1000 cm^−1^ for 2 M ZnSO_4_ electrolyte, pristine ZrOF, and ZrOF wetted in 2 M ZnSO_4_ electrolyte.

Raman spectroscopy (XperRAM C, Nanobase) was utilized to elucidate the solvation structure of Zn^2+^ ions adsorbed on negatively charged ZrOF surface. For this analysis, 2 M ZnSO_4_ electrolyte, pristine ZrOF, and ZrOF wetted in 2 M ZnSO_4_ electrolyte for 24 hours were prepared. In ZnSO_4_ solutions, Zn^2+^ ions generally exist in a hexa-hydrated form, forming a solvent-separated ion pair (SSIP, [Zn^2+^(H_2_O)_6_·SO_4_^2−^]) in the outer solvation shell (Figure S16). Additionally, SO_4_^2−^ ions can participate in the solvation structure of Zn^2+^ ions, leading to the loss of one water molecule and promoting the formation of a contact ion pair (CIP, [Zn^2+^(H_2_O)_5_·OSO_3_^2−^]). The peak area ratio of SSIP and CIP components in 2 M ZnSO_4_ was calculated to be 76% and 24%, respectively, closely aligning with the previously reported values of 77% and 23%.^[S13]^ Furthermore, the *v*(SO_4_^2−^) band of the wetted ZrOF exhibited a noticeable shift to a higher frequency (approximately 989 cm^−1^), suggesting the loss of hydration water from the solvation structure of Zn^2+^ ions. This indicates the negatively charged ZrOF surface changed the solvation structure of Zn^2+^ ions through stronger ionic bonding, leading to the formation of more CIP modes. As noted in Huijun's study, membranes that form strong ionic bonds with hydrated Zn^2+^ ions tend to induce more CIP modes, resulting in a reduction in the number of water molecules bound to the Zn^2+^ ions.^[S14]^


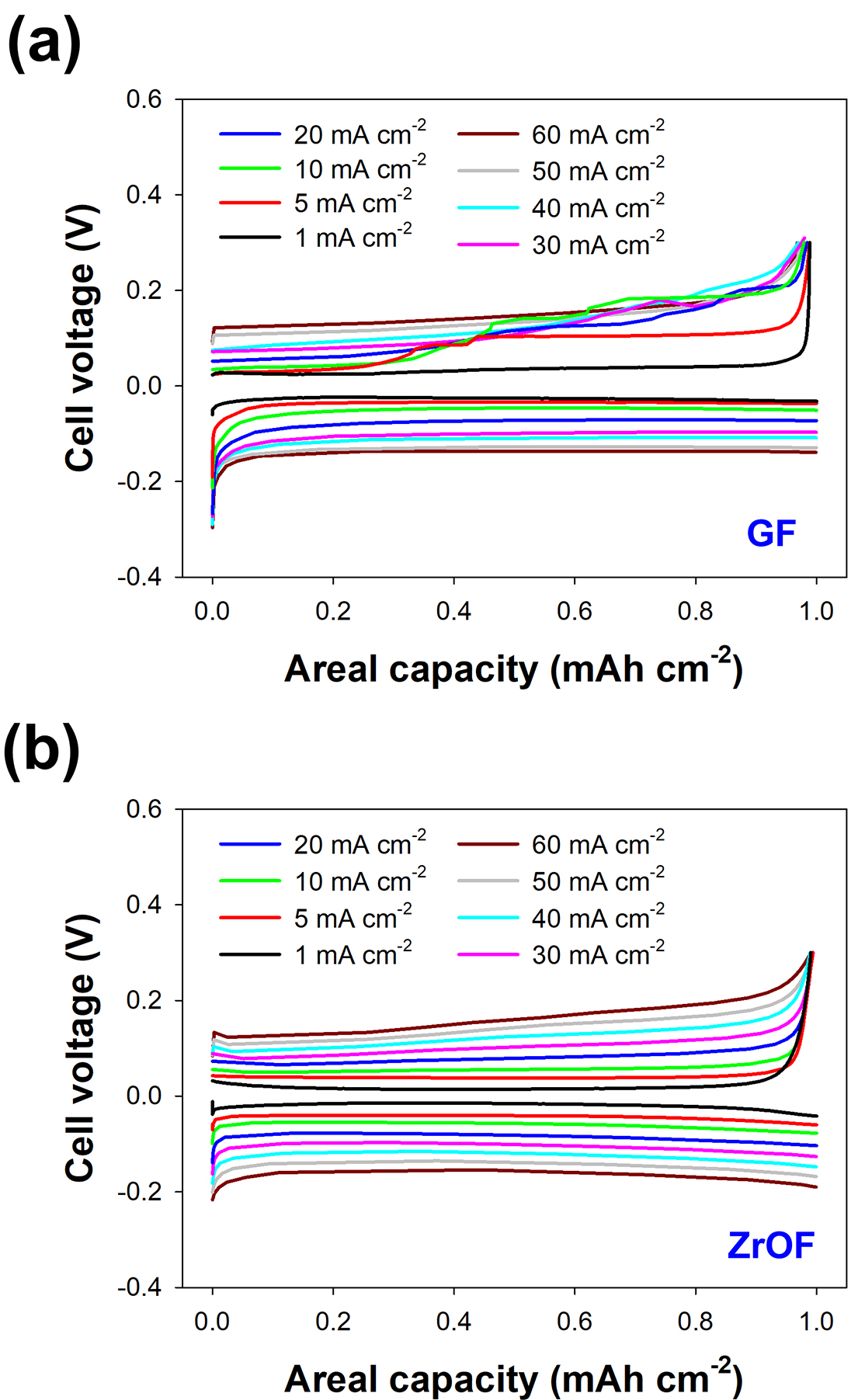


**Figure S17.** Charge-discharge voltage profiles acquired at current densities of 1–60 mA cm^−2^ for carbon/Zn asymmetric cell.


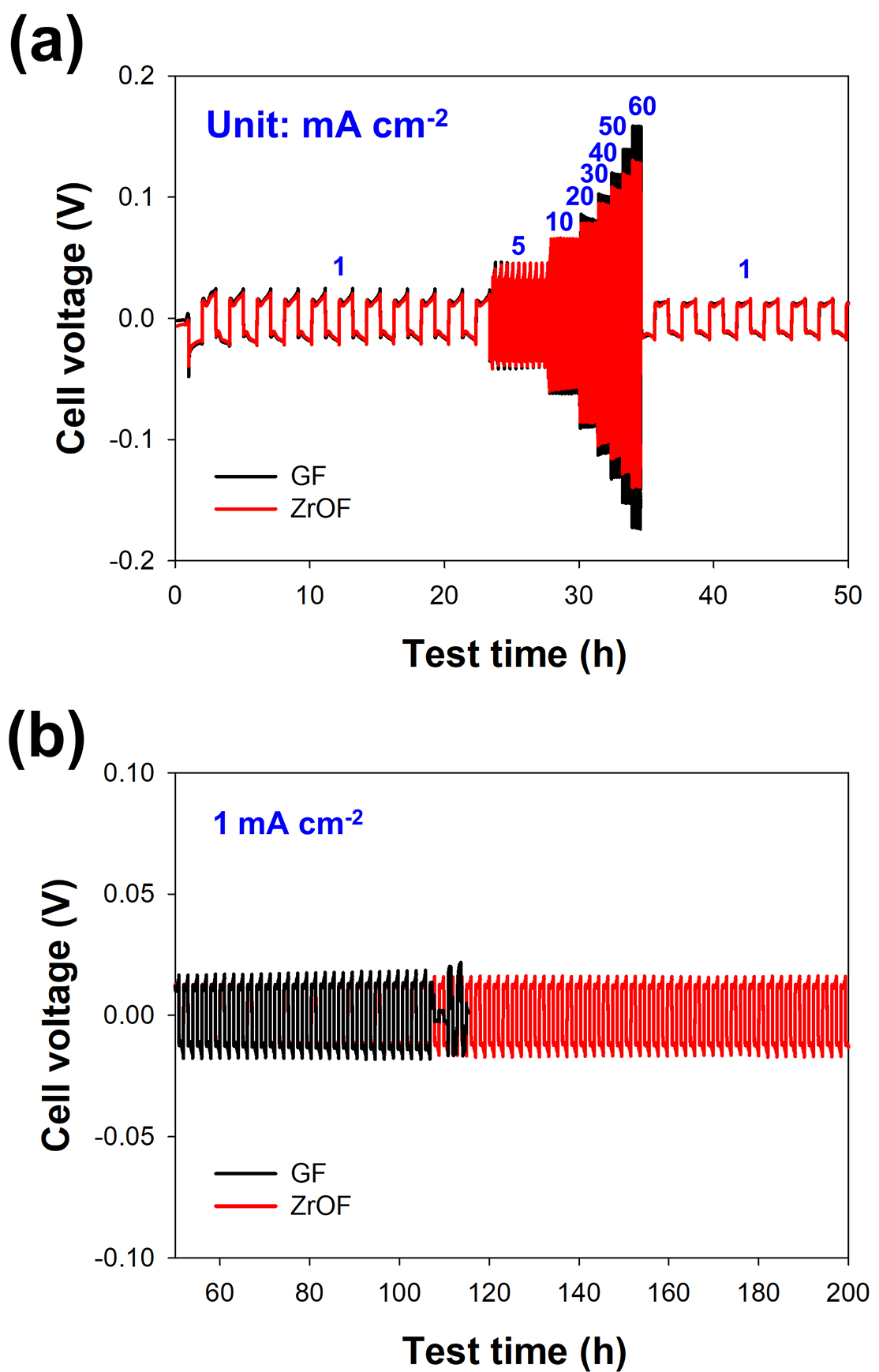


**Figure S18.** a) Voltage-time profiles at the different current densities of 1 – 60 mA cm^−2^ for Zn symmetric cell. b) The cycling performance after returning to operating current density of 1 mA cm^−2^.


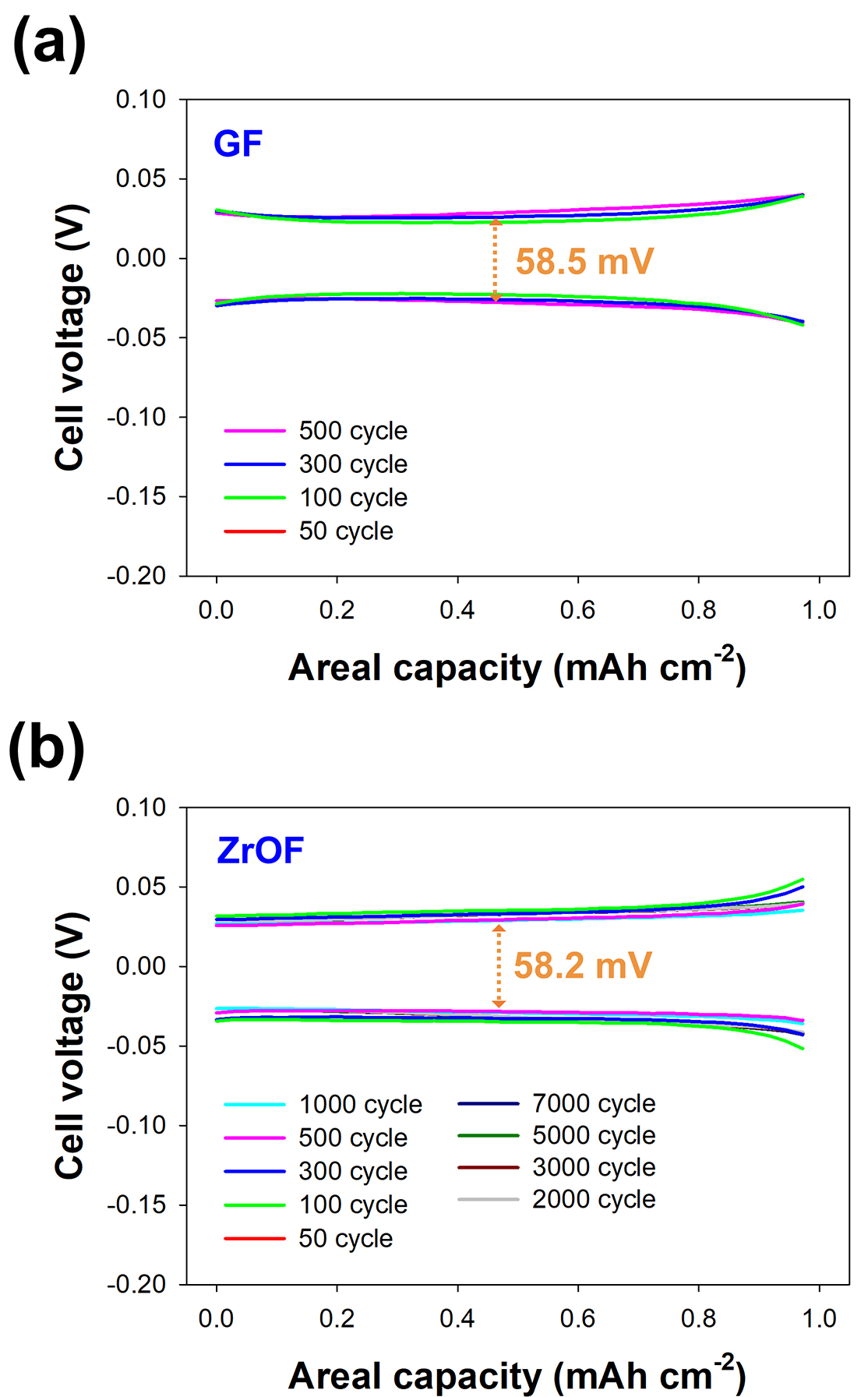


**Figure S19.** Charge–discharge voltage profiles obtained at 1 mAh cm^−2^ and 5 mA cm^−2^ for a) GF and b) ZrOF separators.


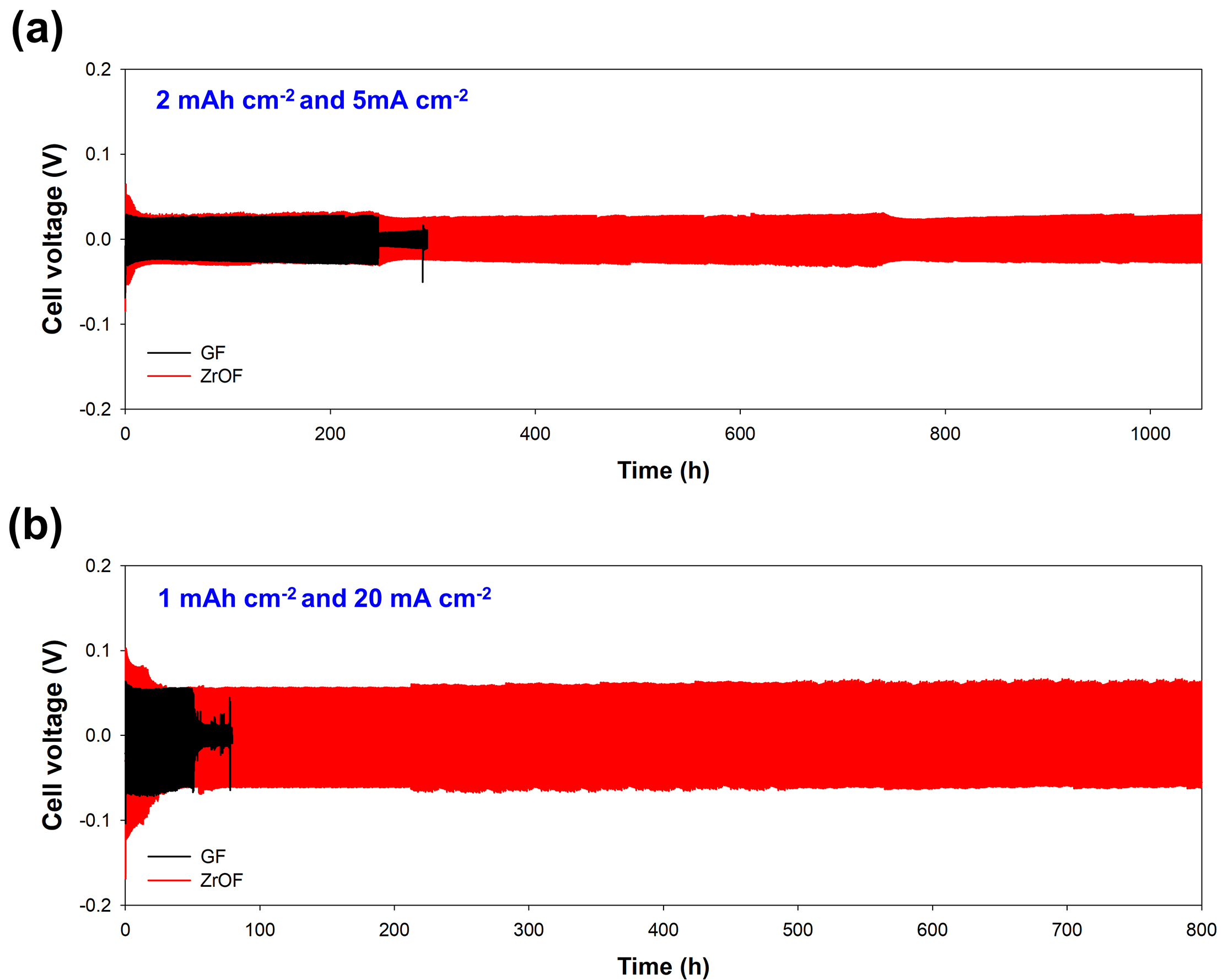


**Figure S20.** Cycling performance of Zn symmetric cell with ZrOF separator under severe operating conditions such as a) 2 mAh cm^−2^/5 mA cm ^−2^ and b) 1 mAh cm^−2^/20 mA cm ^−2^.


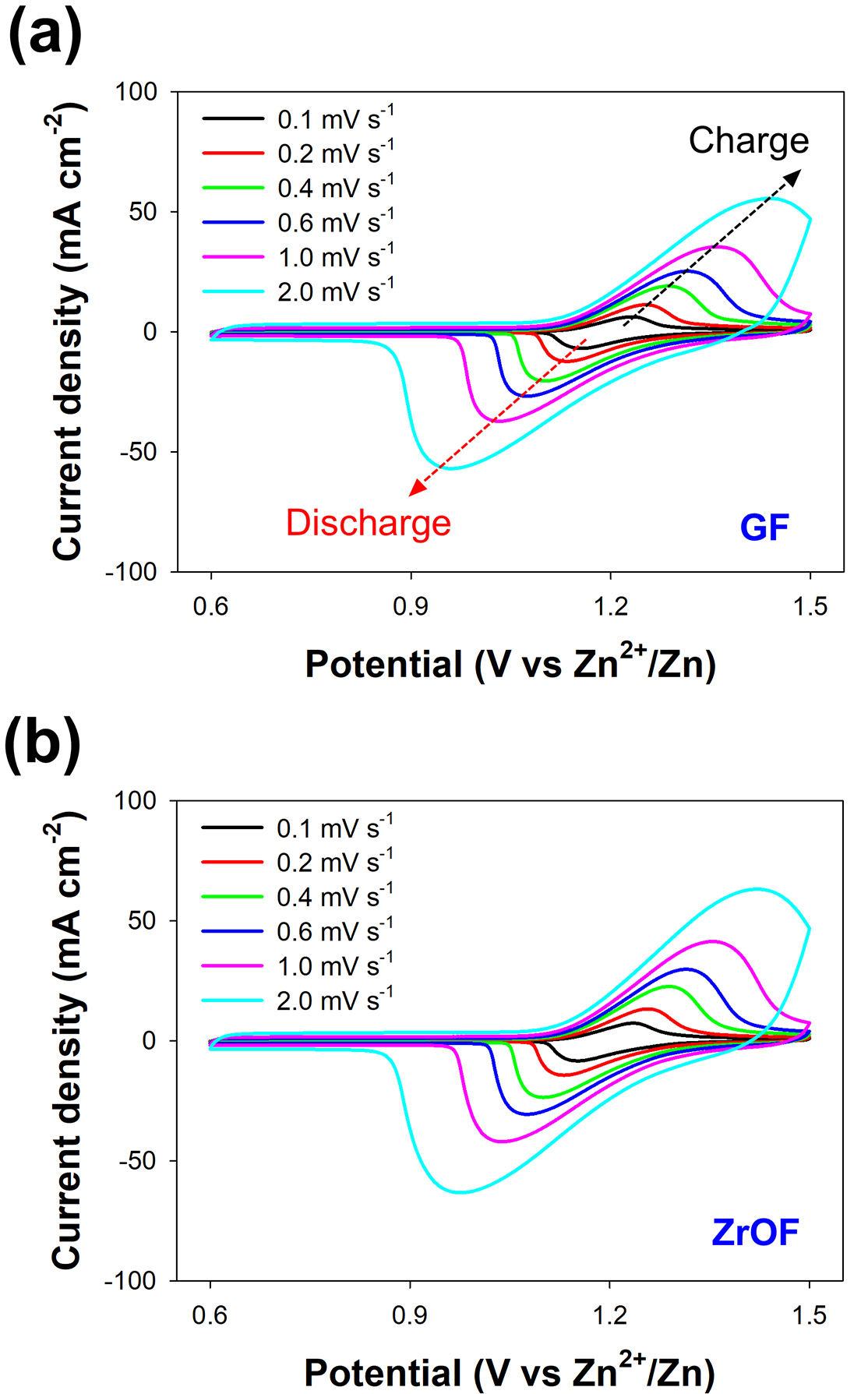


**Figure S21.** CV curves acquired at scan rates of 0.1–2.0 mV s^−1^ for a) GF and b) ZrOF separators.


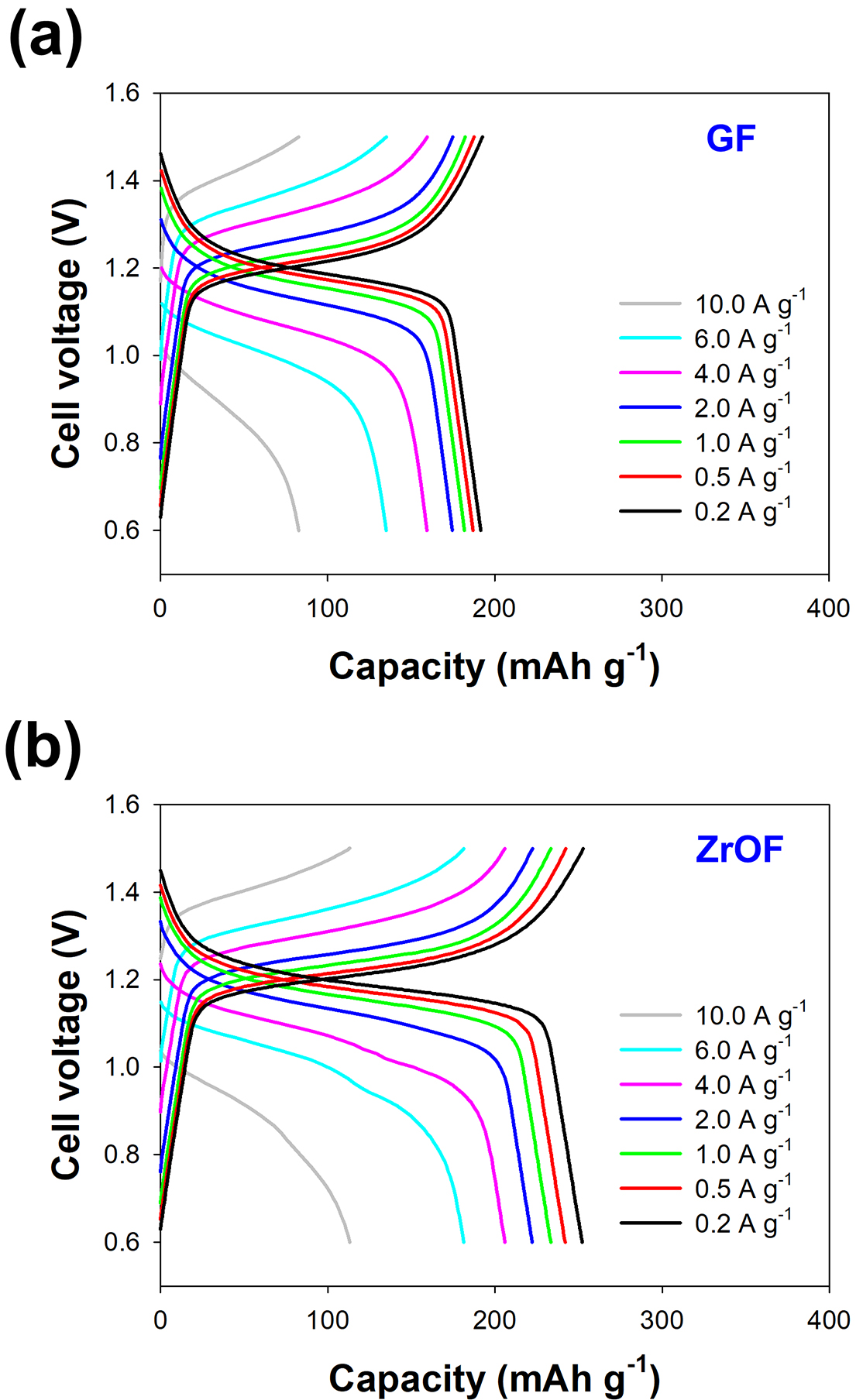


**Figure S22.** Galvanostatic charge–discharge voltage profiles obtained at current densities of 0.2–1.0 A g^−1^ for a) GF and b) ZrOF separators.


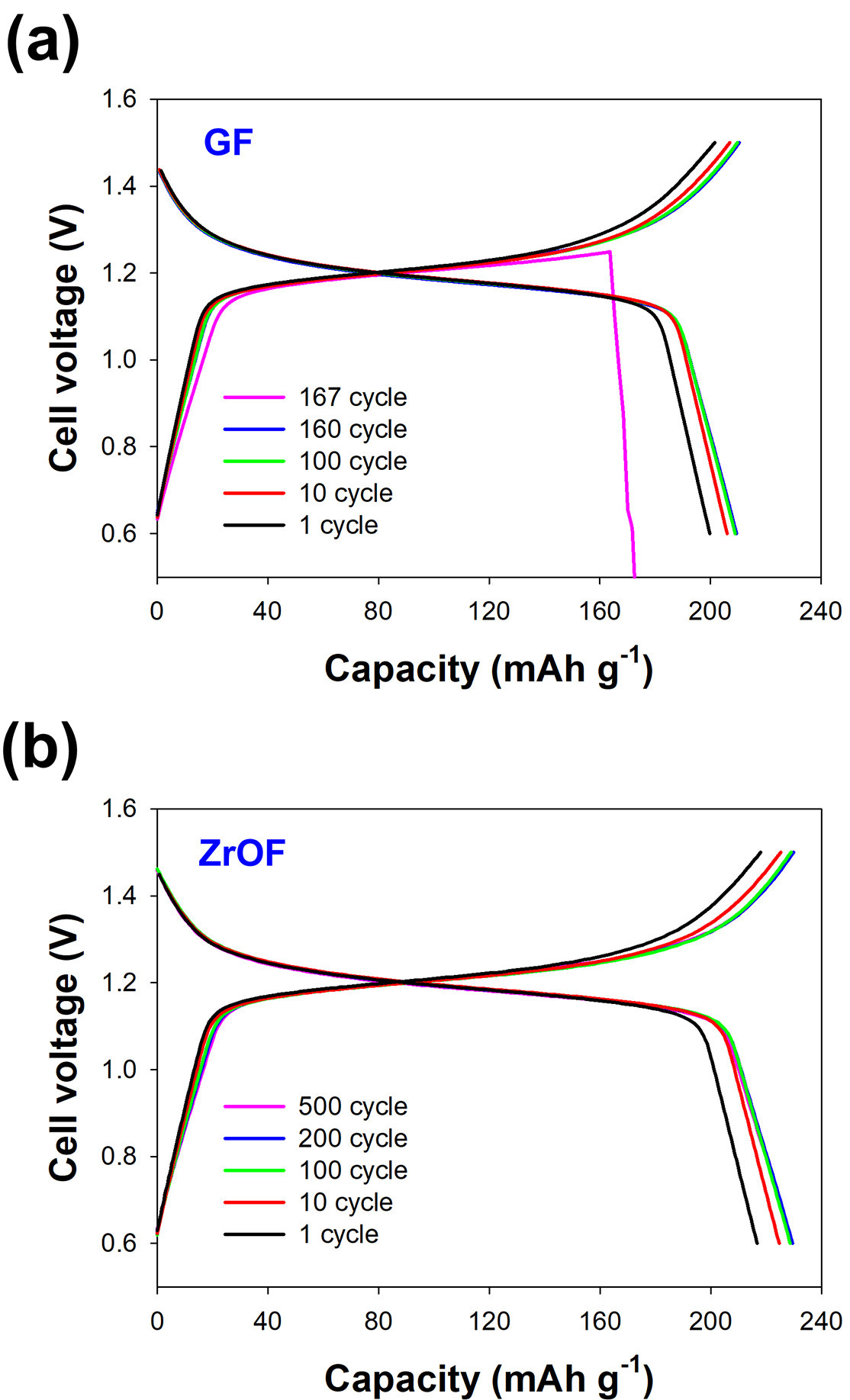


**Figure S23.** Charge–discharge voltage profiles acquired at a constant current density of 0.2 A g^−1^ (2.6 mA cm^−2^) for a) GF and b) ZrOF separators.


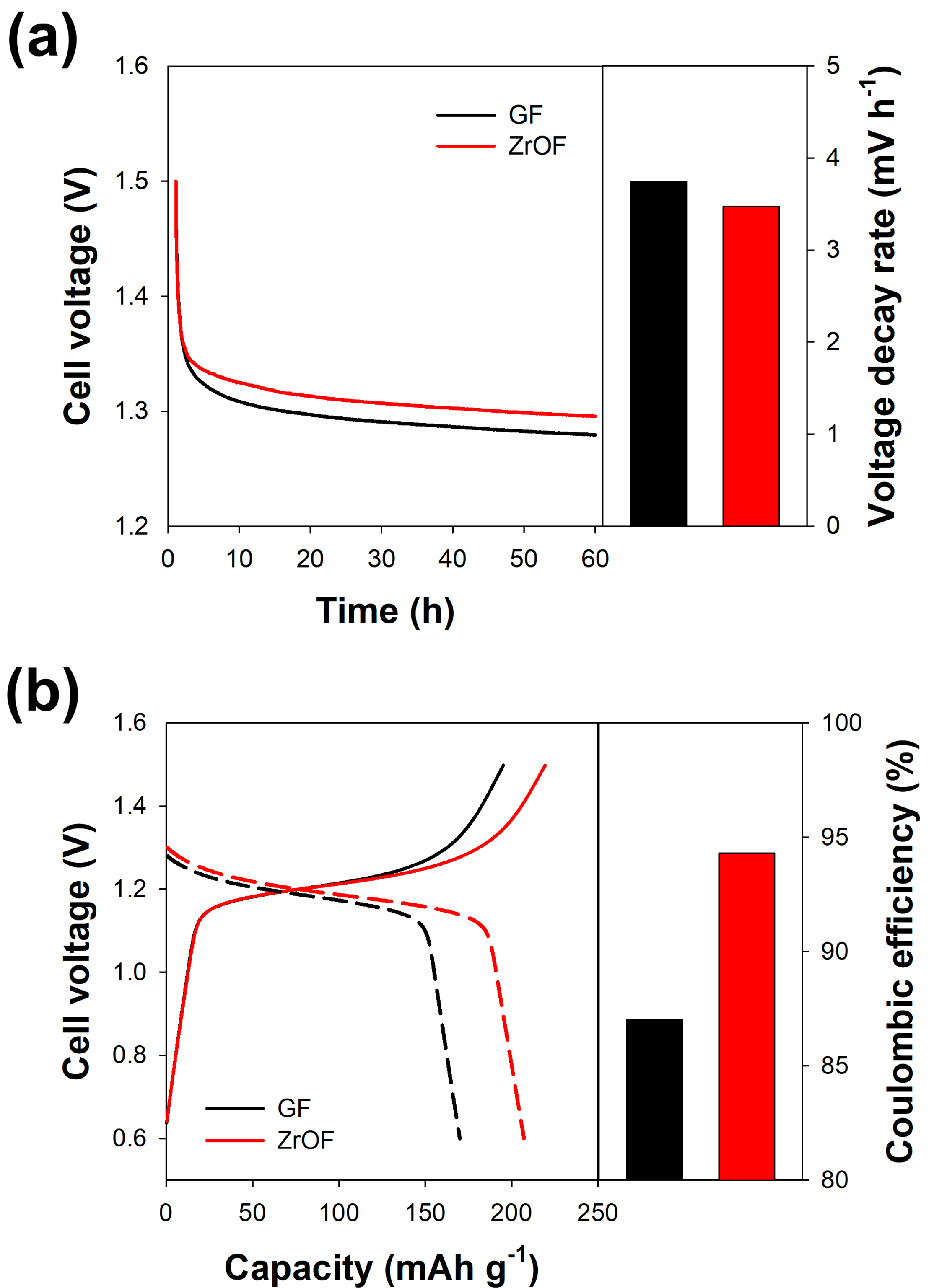


**Figure S24.** a) OCV retention test at 0.2 A g^−1^ for 60 h and corresponding voltage decay rates. b) Self-discharge test after rest time of 48 h and corresponding coulombic efficiency.


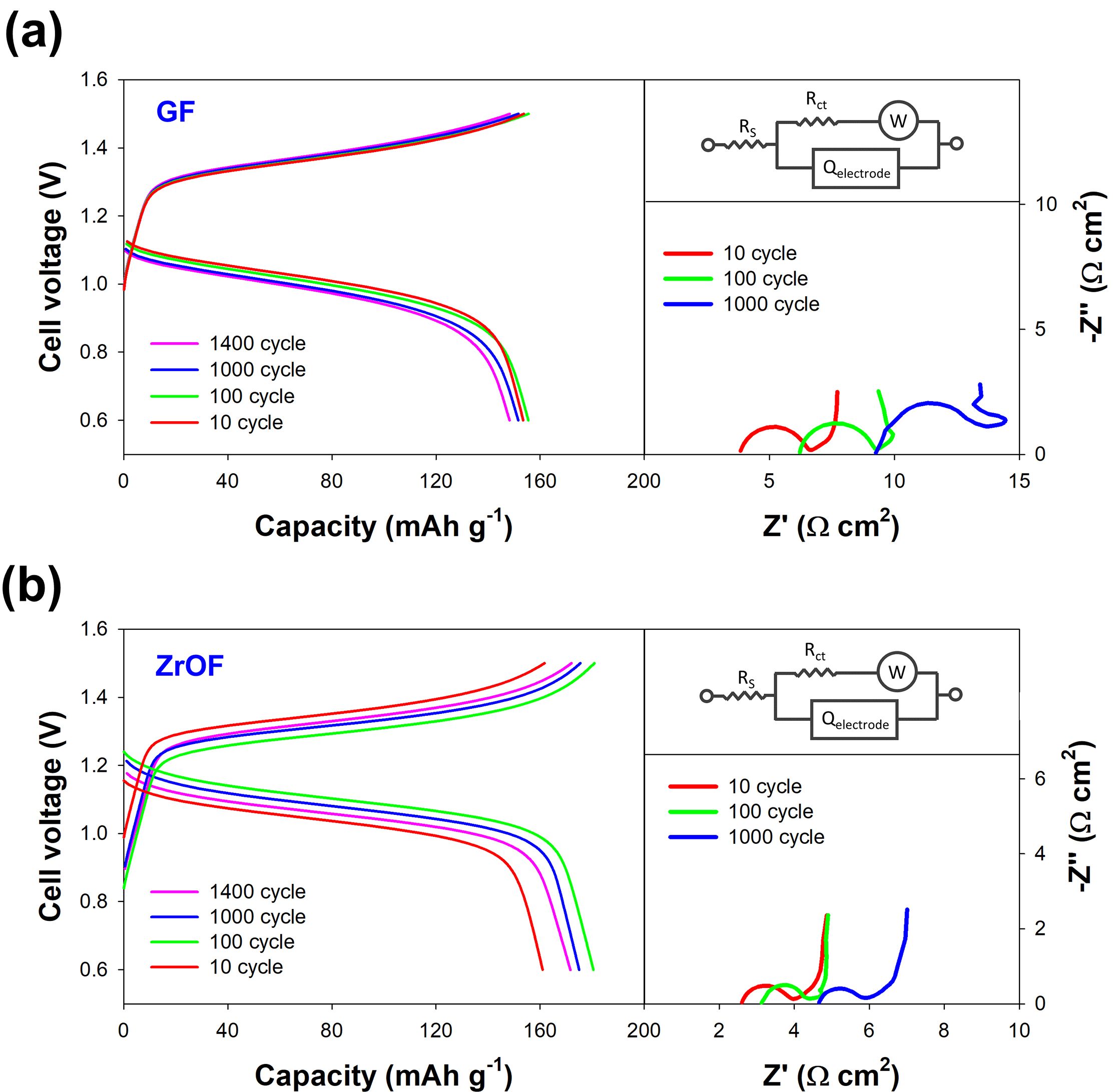


**Figure S25.** Charge–discharge voltage profiles obtained at a constant current density of 4 A g^−1^ (51.5 mA cm^−2^) and corresponding EIS test results after 10th, 100th, and 1000th cycle under OCV conditions in a frequency range of 1 MHz to 10 mHz for a) GF and b) ZrOF separators.


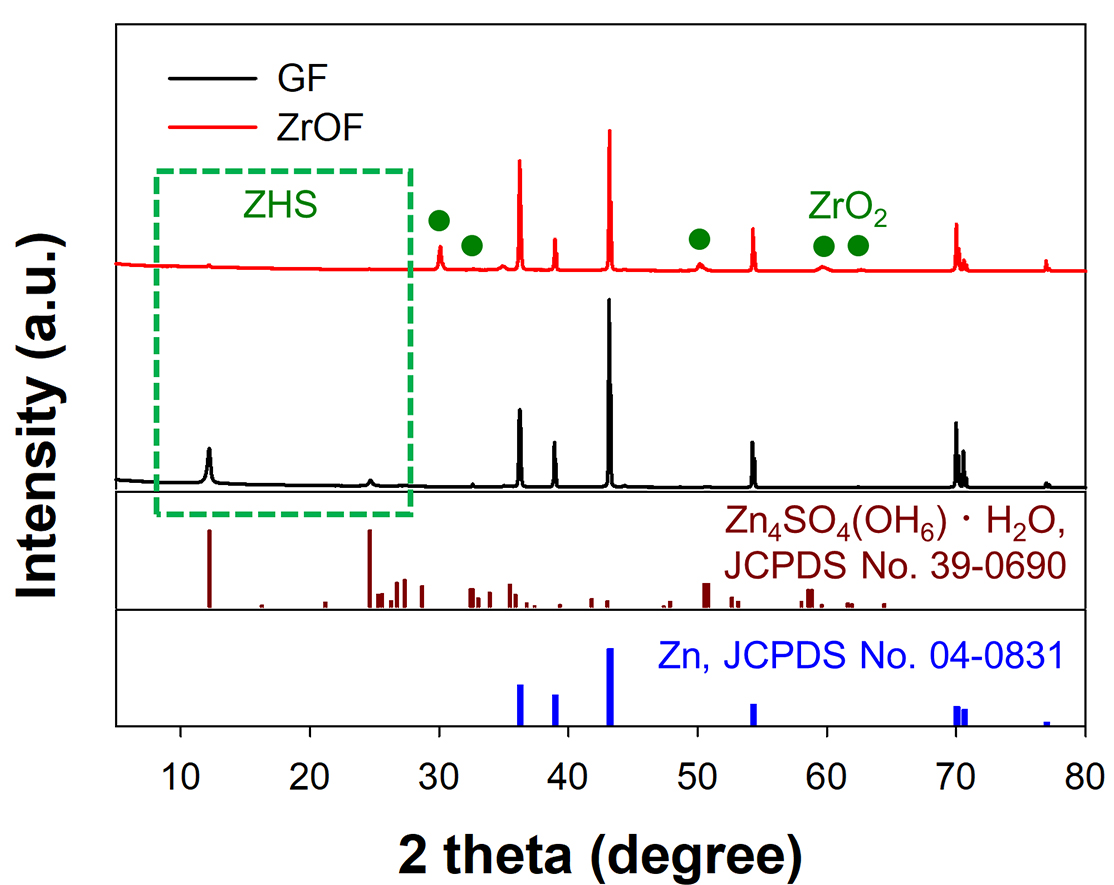


**Figure S26.** XRD patterns of Zn metal electrodes after 1000th discharge cycle.

**
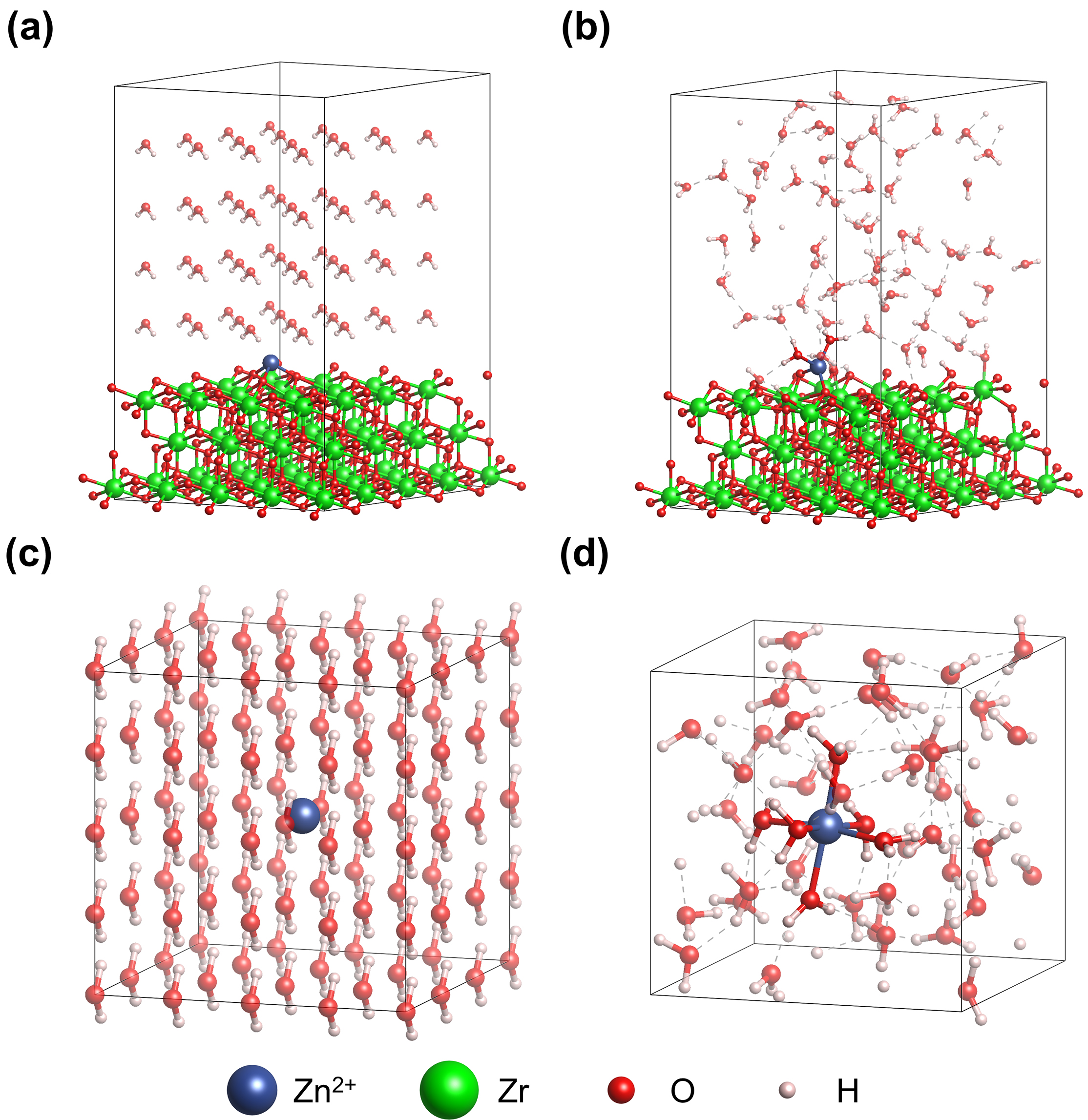
**

**Figure S27.** AIMD snapshots of a Zn^2+^ ion in an ambient H_2_O environment on the ZrO_2_ surface and in bulk solution: (a) Zn^2+^ ion on ZrO_2_ surface at 0.0 ps, (b) Zn^2+^ ion on ZrO_2_ surface at 2.5 ps, (c) Zn^2+^ ion in bulk solution at 0.0 ps, and (d) Zn^2+^ ion in bulk solution at 2.5 ps.

**Table S1.** Cell performance comparison with previously reported Zn–I_2_ batteries.

| Reference | Strategy | Separator | Capacity  (mAh g^−1^) | Current density  C (or A g^−1^) | Number of cycles | Rate of capacity decay  (% cycle^−1^) | |
| --- | --- | --- | --- | --- | --- | --- | --- |
| 71 | Controlling electrolyte-to-iodine rate | Glass fiber or Celgard 3407 | 140 | 2 | 3000 | | 0.003 |
| 72 | Using Pluronic-based gel electrolyte | Cellulose | 210 | 1 | 500 | | 0.011 |
| 73 | Employing N-methyl acetamide-based eutectic electrolyte | Glass fiber | 210 | 2 | 1000 | | NA |
| 74 | Dual plating battery in a water-in-salt electrolyte | Glass fiber | 320 | (0.02) | 500 | | NA |
| 75 | Using ZnHCF film on anode | NA | 220 | 1 | 1000 | | 0.019 |
| 76 | Employing carbon cloth as cathode | Nafion 117 | 275 | (0.2) | 200 | | 0.045 |
| 77 | Using carbon cloth as cathode | NA | 150 | (1.0) | 2000 | | 0.010 |
| 78 | Employing nanoporous activated carbon cloth as cathode | Glass fiber | 160 | 5 | 1500 | | 0.007 |
| 79 | Using ZnCl_2_/KI hydrogel gel as electrolyte | Glass fiber | 100 | 2.0 | 4400 | | 0.005 |
| 80 | Applying biomass-based lignin film to anode | Glass fiber | 183 | 0.4 | 200 | | 0.008 |
| 81 | Using Ti_3_C_2_I_2_ MXene  as cathode | NA | 170 | 3.0 | 2800 | | 0.007 |
| 82 | Pre-embedding I_2_ into Prussian blue as cathode | Glass fiber | 220 | 4.0 | 1000 | | 0.006 |
| 83 | Using Co[Co_1/4_Fe_3/4_(CN)_6_]/I_2_ as cathode | Glass fiber | 166 | 4.0 | 2000 | | 0.010 |
| Present study | Employing ZrOF as separator | ZrO_2_ felt | 230.2 | 1 (0.2) | 500 | | 0.001 |
|  |  |  | 181.6 | 20 (4.0) | 5000 | | 0.004 |

**References**

1. J. H. Park, J. J. Park, O. O. Park, C.-S. Jin, J. H. Yang, *J. Power Sources* **2016**, 310, 137.
2. Y. Song, P. Ruan, C. Mao, Y. Chang, L. Wang, L. Dai, P. Zhou, B. Lu, J. Zhou, Z. He, *Nano-Micro Lett.* **2022**, 14, 218.
3. S. Kim, J. Heo, R. Kim, J. H. Lee, J. Seo, S. Yoon, H. Lee, S. J. Kim, H. T. Kim, *Small* **2021**, 17, 2008059.
4. X. Xie, S. Liang, J. Gao, S. Guo, J. Guo, C. Wang, G. Xu, X. Wu, G. Chen, J. Zhou, *Energy Environ. Sci.* **2020**, 13, 503.
5. N.-U. Seo, K. Kim, J. Yeo, S. J. Kwak, Y. Kim, H. Kim, M. S. Kim, J. Choi, Y. S. Jung, J. Chae, *J. Mater. Chem. A* **2023**, 11, 18953.
6. G. Kresse, J. Furthmüller, *Phys. Rev. B* **1996**, 54, 11169.
7. J. P. Perdew, K. Burke, M. Ernzerhof, *Phys. Rev. Lett.* **1996**, 77, 3865.
8. H. J. Monkhorst, J. D. Pack, *Phys. Rev. B* **1976**, 13, 5188.
9. G. Henkelman, A. Arnaldsson, H. Jónsson, *Comput. Mater. Sci.* **2006**, 36, 354.
10. E. Sanville, S. D. Kenny, R. Smith, G. Henkelman, *J. Comput. Chem.* **2007**, 28, 899.
11. W. Tang, E. Sanville, G. Henkelman, *J. Phys.: Condens. Matter* **2009**, 21, 084204.
12. S. Nosé, *Mol. Phys.* **1984**, 52, 255.
13. X. Bai, Y. Nan, K. Yang, B. Deng, J. Shao, W. Hu, X. Pu, *Adv. Funct. Mater.* **2023**, 33, 2307595.
14. H. Yang, Y. Qiao, Z. Chang, H. Deng, X. Zhu, R. Zhu, Z. Xiong, P. He, H. Zhou, *Adv. Mater.* **2021**, 33, 2102415.
